# Differential requirement of neutralizing antibodies and T cells on protective immunity to SARS-CoV-2 variants of concern

**DOI:** 10.1038/s41541-023-00616-y

**Published:** 2023-02-13

**Authors:** Patrick O. Azevedo, Natália S. Hojo-Souza, Lídia P. Faustino, Marcílio J. Fumagalli, Isabella C. Hirako, Emiliano R. Oliveira, Maria M. Figueiredo, Alex F. Carvalho, Daniel Doro, Luciana Benevides, Edison Durigon, Flávio Fonseca, Alexandre M. Machado, Ana P. Fernandes, Santuza R. Teixeira, João S. Silva, Ricardo T. Gazzinelli

**Affiliations:** 1 Centro de Tecnologia de Vacinas, Universidade Federal de Minas Gerais, Belo Horizonte, Brazil; 2 Instituto René Rachou, Fundação Oswaldo Cruz-Minas, Belo Horizonte, Brazil; 3 Faculdade de Medicina de Ribeirão Preto, Universidade de São Paulo, São Paulo, Brazil; 4 Plataforma Bi-Institucional de Pesquisa em Medicina Translacional - Fiocruz/SP, São Paulo, Brazil; 5 Instituto de Ciências Biológicas, Universidade de São Paulo, São Paulo, Brazil; 6 Instituto de Ciências Biológicas, Universidade Federal de Minas Gerais, Belo Horizonte, Brazil; 7 Faculdade de Farmácia, Universidade Federal de Minas Gerais, Belo Horizonte, Brazil; 8 University of Massachusetts Medical School, Worcester, Massachusetts, USA

**Keywords:** DNA vaccines, Viral infection

## Abstract

The current COVID-19 vaccines protect against severe disease, but are not effective in controlling replication of the Variants of Concern (VOCs). Here, we used the existing pre-clinical models of severe and moderate COVID-19 to evaluate the efficacy of a Spike-based DNA vaccine (pCTV-WS) for protection against different VOCs. Immunization of transgenic (K18-hACE2) mice and hamsters induced significant levels of neutralizing antibodies (nAbs) to Wuhan and Delta isolates, but not to the Gamma and Omicron variants. Nevertheless, the pCTV-WS vaccine offered significant protection to all VOCs. Consistently, protection against lung pathology and viral load to Wuhan or Delta was mediated by nAbs, whereas in the absence of nAbs, T cells controlled viral replication, disease and lethality in mice infected with either the Gamma or Omicron variants. Hence, considering the conserved nature of CD4 and CD8 T cell epitopes, we corroborate the hypothesis that induction of effector T-cells should be a main goal for new vaccines against the emergent SARS-CoV-2 VOCs.

## Introduction

Despite a relatively broad global vaccination program against COVID-19, unequal access to vaccines between high- and low-income countries is a constant threat for emergence of SARS-CoV-2 variants of concern (VOCs). With the appearance of Omicron, we are now facing the fourth pandemic wave^1^. Difficulties in eliminating SARS-CoV-2 and the emergence of VOCs capable of immune evasion have required booster doses. However, even after the fourth dose, the current COVID-19 vaccines fail to control viral replication and to prevent the mild to moderate disease during an Omicron predominant period. This suggests that COVID-19 may become an endemic disease that should be included in the periodic vaccination calendar in several countries^2^.

The race towards the search for vaccines resulted in the development of different vaccine platforms, with varying levels of efficacy and effectiveness^3^. The third generation of vaccines employ the strategy to transfer to the host, nucleic acids encoding antigens that are then expressed by the host cells. mRNA-based vaccines against COVID-19 have shown high efficacy and have been widely used in the world^4^. In addition, vaccines based on non-replicating adenovirus vectors such as recombinant human adenovirus type 26 (Ad26) and chimpanzee adenovirus modified (ChadOx1) are effective and have been distributed in different countries. These vaccines encoding the SARS-CoV-2 Spike (S) protein induce strong humoral and cellular responses, however, frequent booster doses are required to protect against the emergent VOCs^5^. DNA-based vaccines, still under development, have shown promising and consistent results in stimulating both humoral and cellular immune responses^6,7^. It is likely that DNA vaccines may also require booster doses, but they have other advantages, such as low cost, high stability at room temperature and of easy distribution^8^. Furthermore, this platform allows for an immediate response in the case of emergent endemics/pandemics of pathogens with high rate of transmission.

Accumulating data highlighted the importance of T cells in mediating immunity to SARS-CoV-2^9–12^, including for Beta^13^, Alpha and Delta^14^ and Omicron^15,16^ VOCs. Individuals vaccinated with adenovirus or mRNA vaccine and COVID-19 convalescents show low levels of neutralizing antibodies (nAbs), but a robust cross-reactive of CD4 and CD8 T cell response against the VOCs, including the most distinct Omicron variant. These findings suggest that protection from severe disease, despite of low levels of VOC-specific nAbs, is largely mediated by T cells^17–22^.

In this context, a DNA-based platform that elicit a strong protective immune response mediated by T cell response is an important strategy to rapidly adapt the COVID-19 vaccines to the new VOCs. Here we tested an experimental vaccine using a plasmid DNA containing the sequence encoding the S protein of the original Wuhan SARS-CoV-2 (pCTV-WS) and evaluated the efficacy to the VOCs in the transgenic mouse (K18-hACE2) and hamster models of severe and moderate human disease, respectively. Our results show that this vaccine induces high, intermediate and low levels nAbs to Wuhan, Delta and Gamma variants, respectively, but no nAbs to the Omicron isolates. Immunization with the pCTV-WS vaccine also induced a robust T cell response to the WS protein. Protective immunity elicited by this vaccine was primarily mediated by nAbs and T cells for the Wuhan/Delta and Gamma/Omicron variants, respectively. Considering the conserved CD4 and CD8 T cell epitopes, the second generation of PAM-SARS-CoV-2 vaccines should aim the induction of robust T-cell responses.

## Results

### Construction of SARS-CoV-2 DNA vaccine

**Fig. 1:**
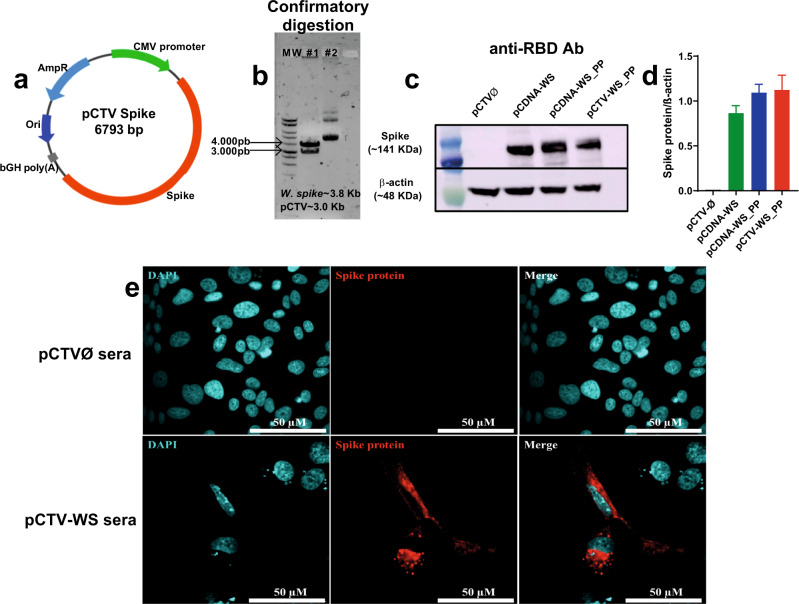
Generation of plasmid pCTV-WSpike (pCTV-WS). **a** Schematic representation of plasmid pCTV-WS used in immunogenicity and protection assays against COVID-19. **b** Confirmatory digestion showing the presence of a ~3.8 Kb fragment corresponding to gene sequence of S protein from SARS-CoV-2 and the pCTV vector with approximate size of ~3.0 Kb. **c**, **d** Representative western blot showing WS protein expression in HEK-293 cells after transfection with pCTVØ (black bar), pCDNA-WS (green bar), pCDNA-WS_PP (blue bar) and pCTV-WS_PP (red bar). PP proline mutation. **e** Immunofluorescence performed with Vero E6 cells transfected with pCTV-WS and incubated with sera from mice immunized with pCTVØ (upper panel) or pCTV-WS (lower panel). Data pooled from two independent experiments. The statistical analysis was performed using One-Way ANOVA followed by Bonferroni post-hoc test. Bars represent mean ± SEM.

The codon-optimized DNA sequence encoding full-length SARS-CoV-2 Spike (S) gene from Wuhan-Hu-1 isolate (WS) (GenBank accession number: MN908947) was synthesized and cloned in the pCTV vector, being named pCTV-WS (Fig. 1a). The WS sequence also contains a mutation (_PP) at the S1/S2 cleavage site that confers stabilization in the pre-fusion conformation. Restriction digestion analyses demonstrated the presence of the 3813 bp fragment of the WS gene in pCTV-WS (Fig. 1b). To compare the expression of the WS protein using different plasmids, HEK293 cells were transfected with the empty vector (pCTVØ), a commercial plasmid containing the full-length WS gene sequence of original SARS-CoV-2 isolated at Wuhan, China (pcDNA-WS), a commercial plasmid containing the codon-optimized and mutated sequence of SARS-COV-2 full-length S gene (pcDNA-WS_PP) and a plasmid derived from pCDNA3.1 containing the codon-optimized and mutated SARS-COV-2 full-length WS gene (pCTV-WS_PP). Neither codon optimization or the mutation altered the S protein expression by HEK293 cells. Moreover, the pCTV vector showed similar expression levels, when compared to pCDNA (Fig. 1c, d). Vero E6 cells were transfected with pCTV-WS and an immunofluorescence was performed using sera samples from mice immunized with pCTVØ or pCTV-WS. Expression of S protein by transfected cells was only observed with sera from pCTV-WS immunized mice (Fig. 1e).

### pCTV-WS immunization induces strong humoral response

**Fig. 2:**
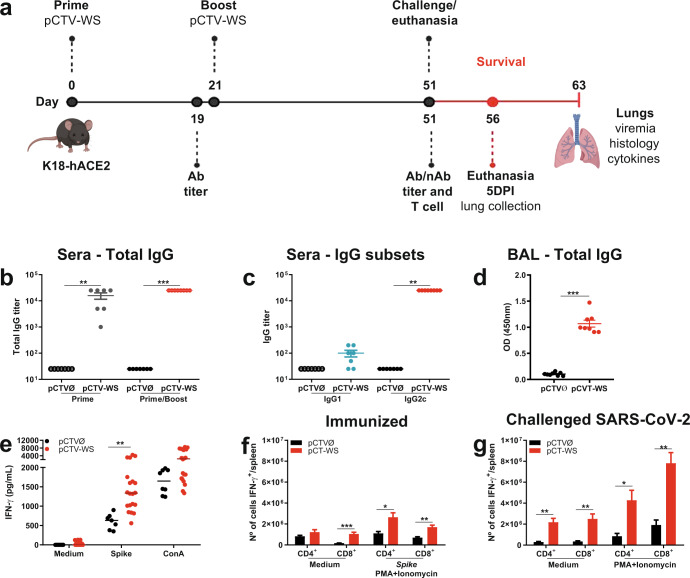
pCTV-WS immunogenicity and protection in immunized K18-hACE2 mice. **a** Schematic representation of the experimental design used to assess humoral and cellular immune response and protection against SARS-CoV-2. **b** Anti-WS total IgG titer in pCTVØ (open circles) or pCTV-WS (gray circles) immunized mice after 1^st^ immunization (prime) and pCTVØ (filled circles) or pCTV-WS (red circles) after prime/boost [*n* = 8 mice/group]. **c** IgG1 (blue circles) and IgG2c (red circles) subclass 30 days after prime/boost pCTV-WS immunization. IgG1 (open circle) and IgG2c (filled circle) in the control group [n = 8 mice/group]. **d** Anti-WS total IgG in bronchoalveolar lavage (BAL) from mice immunized with pCTVØ (black circles) or pCTV-WS (red circles) [*n* = 8 mice/group]. **e** IFN-γ levels produced by splenocytes from pCTVØ (black circles) or pCTV-WS (red circles) immunized mice after 72 h of incubation with medium (negative control), restimulation with recombinant WS protein or Concanavalin A (positive control) [*n* = 8, pCTVØ and *n* = 20, pCTV-WS]. IFN-γ production by CD4^+^ T cells and CD8^+^ T cells from immunized mice stimulated in vitro with WS protein **f** or from mice challenged with Wuhan strain at 5DPI **g** (*n* = 4 for both groups). Data pooled from two independent experiments. The statistical analysis of IgG measured in BAL and IFN-γ measurements was performed using unpaired t-test or Mann-Whitney U test, according to data distribution. Bars represent mean ± SEM. *p < 0.05; **p < 0.01; ***p < 0.001.

K18-hACE2 transgenic mice were immunized via i.m. with two doses containing 100 μg of pCTV (Ø or WS) three weeks apart. Antibody titers were measured before the second dose and 30 days later (Fig. 2a). T cell response was also evaluated 30 days after the second dose. Four/five animals from each group were challenged with SARS-CoV-2 and followed for 12 days for survival or killed at 5 days post-infection (DPI) for determination of viral load, inflammatory profile and histopathological analysis (Fig. 2a). After pCTV-WS first dose (prime), mice developed significant levels of total IgG anti-WS (Fig. 2b—gray circles). These levels were increased 30 days after the second dose (boost) (Fig. 2b—red circles). When the levels of IgG isotypes were determined, we observed that IgG2c was predominant in mice immunized with pCTV-WS (Fig. 2c). Individual antibody curves for IgG subsets are presented in Supplementary Fig. 1a, b. Significant levels of total IgG anti-WS were also found in the bronchoalveolar lavage fluid (BAL) (*p* < 0.001) (Fig. 2d).

In addition to the strong humoral immune response, a significant cellular response was also observed. Splenocytes from pCTV-WS immunized animals produced IFN-γ in response to restimulation with WS protein in comparison to control group (*p* < 0.01) (Fig. 2e). Mice immunized and then challenged with SARS-CoV-2 showed a higher number of CD4^+^ and CD8^+^ T cells-producing IFN-γ compared to CD4^+^ and CD8^+^ T cells from immunized mice restimulated in vitro with S protein from the Wuhan isolate (Fig. 2f, g).

### pCTV-WS immunization protects against Wuhan strain and reduces viral load

**Fig. 3:**
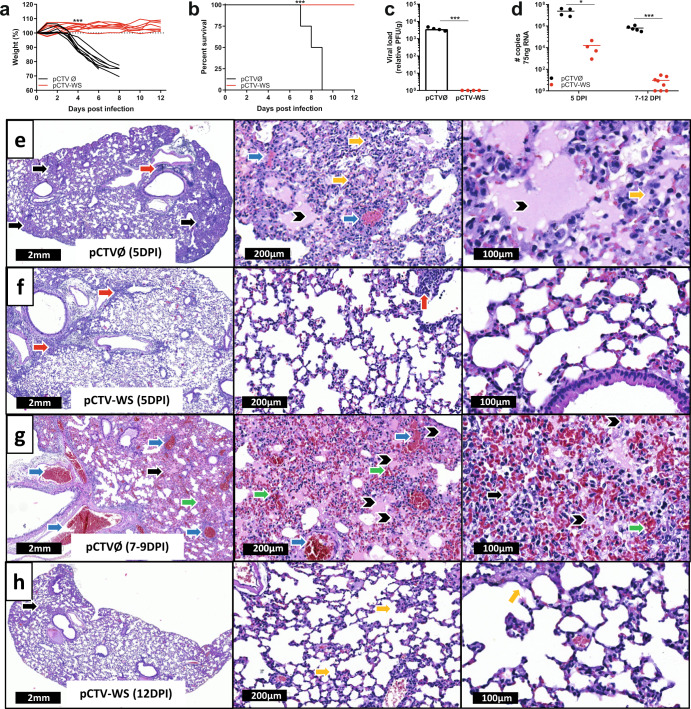
Protection and viral load in pCTV-WS immunized K18-hACE2 mice. Body weight **a** and survival **b** of pCTVØ (black lines) or pCTV-WS (red lines) immunized mice and challenged with the Wuhan strain of SARS-CoV-2 [*n* = 8 mice/group]. **c** Lung viral load in pCTVØ (black bar) or pCTV-WS (red bar) immunized K18-hACE2 mice and challenged with the Wuhan strain at 5 DPI [*n* = 4 mice/group]. **d** SARS-CoV-2 RNA copy number in pCTVØ (black circles) or pCTV-WS (red circles) immunized K18-hACE2 mice and challenged with the Wuhan strain at 5 and 7-12 DPI [*n* = 4–5, pCTVØ and *n* = 4–8 pCTV-WS]. Histopathological sections of lungs from pCTVØ **e**, **g** and pCTV-WS **f**, **h** immunized K18-hACE2 mice at 5 DPI and 7–12 DPI, respectively [*n* = 4 mice/group]. Inflammatory infiltrate (black arrow), perivascular inflammatory infiltrate (red arrow), alveolar septal thickening (yellow arrow), vascular congestion (blue arrow), edema (arrow head) and hemorrhage (green arrow) at 1.1×, 10×, and 20× magnification. The statistical analysis of weight curves was performed by area under the curve followed by unpaired t-test. The statistical analysis of survival was performed using the log-rank test. The statistical analysis of viral load was performed using unpaired t-test. *p < 0.05; ***p < 0.001.

All animals immunized with pCTV-WS and challenged with SARS-CoV-2 maintained the body weight and survived a challenge infection. On the other hand, all animals in the control group lost weight at 4 DPI, and died up to 8 DPI (*p* < 0.001) (Fig. 3a, b). Viral load was measured in lung samples at 5 DPI. Immunized animals showed no PFU while the control group exhibited infection rates greater than 10^3^PFUs/g of tissue (Fig. 3c). Despite the absence of viable virus in immunized animals, lower levels of viral RNA were found in lung samples at 5 DPI (*p* < 0.05) and 7–12 DPI (*p* < 0.001) in comparison to control group (Fig. 3d). Histopathological analysis of lung samples from non-immunized mice (pCTVØ) euthanized at 5 DPI showed loss of alveolar architecture with an intense and diffuse inflammatory infiltrate (black arrow), alveolar septum thickening (yellow arrow), edema (arrowhead) and vascular congestion (blue arrow) (Fig. 3e). On the other hand, pCTV-WS immunized animals showed only perivascular inflammatory infiltrate (red arrow) with preserved lung parenchyma (Fig. 3f). At 7–9 DPI pCTVØ mice exhibited an intense and diffuse inflammatory infiltrate (black arrow) associated with vascular congestion (blue arrow), hemorrhage (green arrow) and edema (black arrowhead) (Fig. 3g). pCTV-WS mice at 12 DPI presented alveolar areas preserved with some inflammatory focus (black arrow) and alveolar septum thickening (yellow arrow) (Fig. 3h). Mice normal lung tissue sections can be observed in Supplementary Fig. 2a.

### pCTV-WS immunization reduces inflammation and induces the recruitment of B and T cells into the lungs

**Fig. 4:**
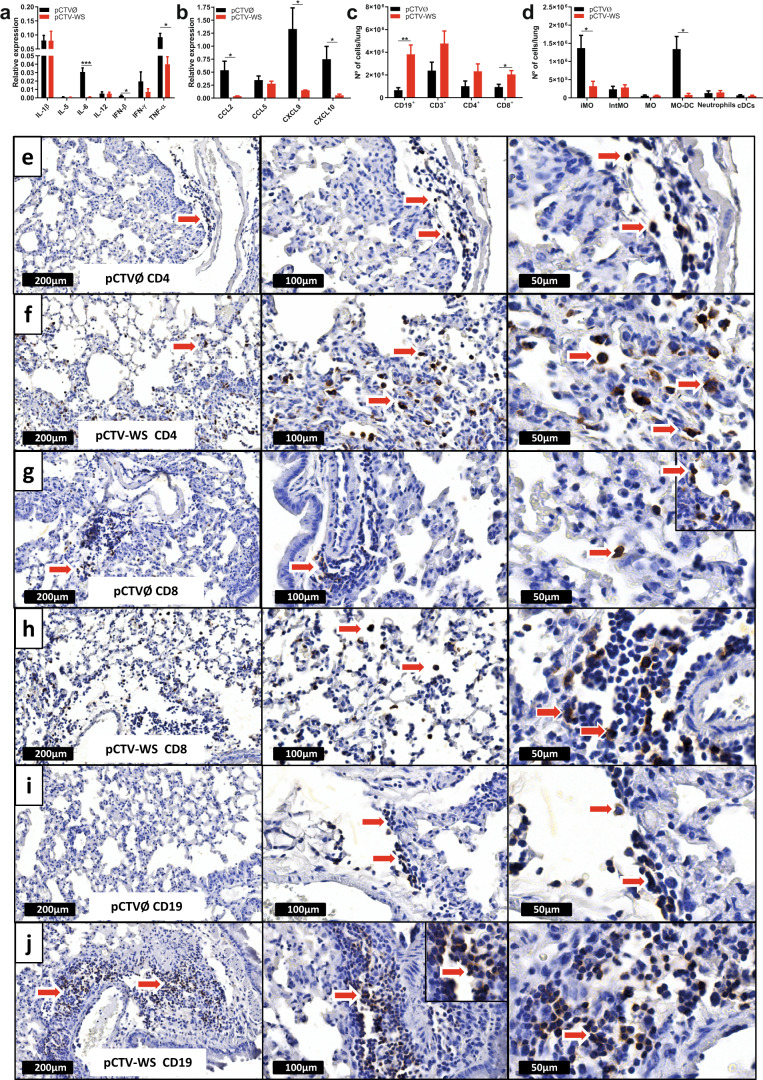
Inflammatory profile and immunohistochemical staining of B and T cells in lung samples from pCTV-WS immunized K18-hACE2 mice. Relative expression of cytokines **a** and chemokines **b** in lung samples of pCTVØ (black bar) or pCTV-WS (red bar) immunized mice and challenged with the Wuhan strain at 5 DPI [*n* = 4 mice/group]. Absolute number of B cells (CD19^+^), T cells (CD3^+^), CD4^+^ and CD8^+^ T cells **c** and inflammatory monocytes (F4/80^+^CD11b^+^Ly6C^high^), intermediate monocytes (F4/80^+^CD11b^+^Ly6C^int^), monocytes (F4/80^+^CD11b^+^Ly6C^-^), monocyte-derived dendritic cells (F4/80^+^CD11b^+^Ly6C^high^DC-Sign^+^), neutrophils (F4/80^-^CD11b^high^Ly6G^+^) and classical dendritic cells (CD11b^-/low^CD11c^+^MHC-II^+^) **d** at 5 DPI in lung samples of pCTVØ (black bar) and pCTV-WS (red bar) immunized K18-hACE2 mice, by flow cytometry [*n* = 4 mice/group]. Immunohistochemical staining of lungs tissue sections from pCTVØ and pCTV-WS mice challenged with Wuhan strain stained with anti-CD4 **e**, **f**, anti-CD8 **g**, **h**, and anti-CD19 **i**, **j** showing the localization of CD4^+^ T cells, CD8^+^ T cells and B cells (red arrows) in lung tissue at 10×, 20×, and 40× magnification. The statistical analysis was performed using unpaired t-test. Bars represent mean ± SEM. *p < 0.05; **p < 0.01; ***p < 0.001.

A pronounced inflammatory reaction was observed in lung samples from the control group, as indicated by the presence of inflammatory cytokines as IL-6, IFN-β and TNFα as well as chemokines as CCL2, CXCL9 and CXCL10 in higher levels in the control group compared to immunized animals (Fig. 4a, b). pCTV-WS immunized animals showed an increase in the number of B cells (CD19^+^) and CD8^+^ T cells in the lungs at 5 DPI (Fig. 4c). Differently, the control group exhibited a strong inflammatory reaction with inflammatory monocytes and DC’s derived from monocytes (MO-DC) (Fig. 4d). The immunohistochemical analyses showed CD4^+^ and CD8^+^ T cells diffuse in the lung tissue (Fig. 4e–h) and B cells (CD19^+^) more restricted to the perivascular region (Fig. 4i, j).

### pCTV-WS immunization protects against SARS-CoV-2 variants of concern in severe COVID-19 experimental model

**Fig. 5:**
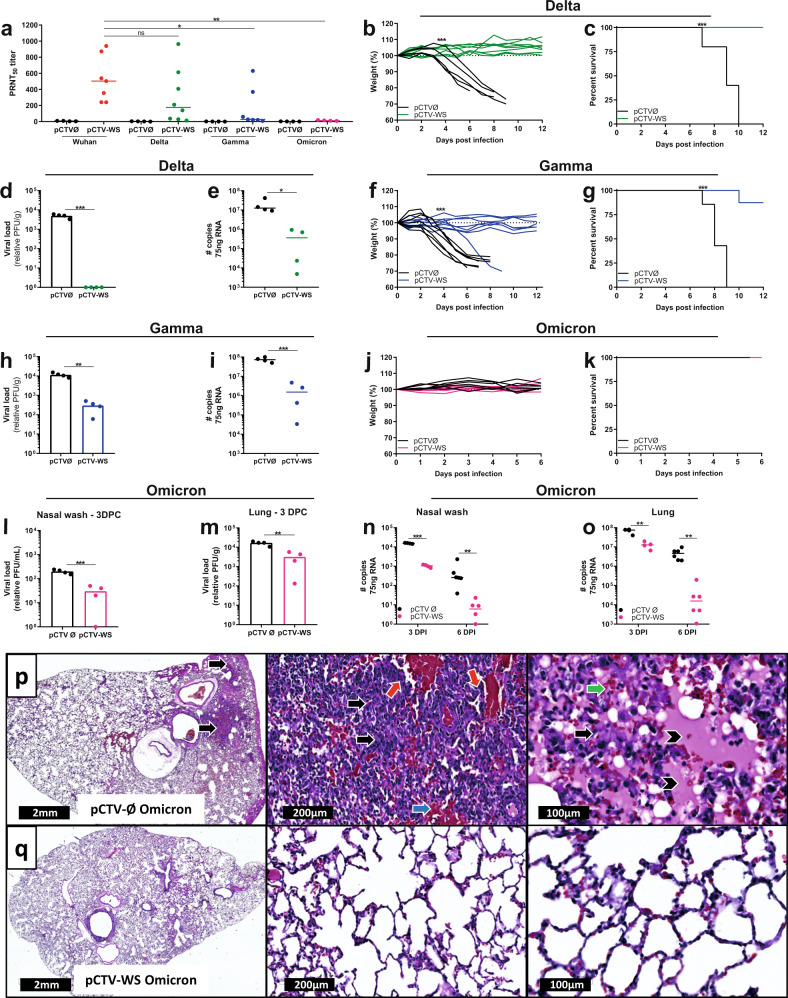
Protection against SARS-CoV-2 variants of concern in pCTV-WS immunized K18-hACE2 mice. **a** PRNT50 was evaluated using different SARS-CoV-2: Wuhan strain (B lineage), Delta, Gamma and Omicron variants in sera samples from pCTVØ (black circles) or pCTV-WS immunized K18-hACE2 mice (colored circles) [*n* = 4, pCTVØ and *n* = 4–8, pCTV-WS]. Body weight **b** and survival **c** of pCTVØ (black lines) or pCTV-WS immunized K18-hACE2 mice challenged with Delta (green lines). Viable viral load **d**, and SARS-CoV-2 RNA copy number **e** at 5DPI in pCTVØ (black circles) and pCTV-WS immunized mice challenged with Delta variant (green circles). [*n* = 4–6, pCTVØ and *n* = 4–8 pCTV-WS]. Body weight **f** and survival **g** of pCTVØ (black lines) or pCTV-WS immunized K18-hACE2 mice challenged with Gamma variant (blue lines). Viable viral load **h** and SARS-CoV-2 RNA copy number **i** at 5DPI in pCTVØ (black circles) and pCTV-WS immunized mice challenged with Gamma variant (blue circles). [*n* = 4–6, pCTVØ and *n* = 4–8 pCTV-WS]. Body weight **j** and survival **k** of pCTVØ (black lines) or pCTV-WS immunized K18-hACE2 mice challenged with Omicron variant (pink lines). Viral load in nasal wash **l** and lung **m** of pCTVØ (black bar) or pCTV-WS (pink bar) immunized K18-hACE2 mice and challenged with Omicron variant at 3 DPI [*n* = 4 mice/group]. SARS-CoV-2 RNA copy number in nasal wash **n** and lung **o** of pCTVØ (black circles) or pCTV-WS (pink circles) immunized K18-hACE2 mice and challenged with Omicron variant at 3 and 6 DPI [*n* = 4–6mice/group]. Histopathological sections of lungs from pCTVØ **p** or pCTV-WS **q** immunized K18-hACE2 mice and challenged with Omicron variant at 6 DPI. Inflammatory infiltrate (black arrow), perivascular inflammatory infiltrate (red arrow), vascular congestion (blue arrow), edema (arrow head) and hemorrhage (green arrow) at 1.1×, 10×, and 20× magnification. Data pooled from one or two independent experiments. The statistical analysis of weight curves was performed by area under curve followed by unpaired t-test or Mann-Whitney U test, according to the data distribution. The statistical analysis of survival was performed using the log-rank test. The statistical analysis of viral load and RNA copy number was performed using unpaired t-test or Mann-Whitney U test, according to data distribution. *p < 0.05; **p < 0.01; ***p < 0.001.

Neutralizing antibodies against different SARS-CoV-2 variants were evaluated in sera samples of pCTV-WS immunized mice (Supplementary Fig. 1c–f). The PRNT50 titer was around 500 for Wuhan strain (Fig. 5a). Despite the reduction in PRNT50 titer for Delta variant, it was not statistically significant. A drastic reduction in neutralization capacity was observed for the Gamma and Omicron variants, with no animal showing neutralizing titer for Omicron (Fig. 5a).

pCTV-WS immunized mice were also challenged with Delta, Gamma and Omicron variants. pCTV-WS protected 100% of animals challenged with Delta variant (Fig. 5b, c). These animals did not lose weight (*p* < 0.001) and showed 100% survival (*p* < 0.001), compared to 100% mortality in the control group. Immunized mice and challenged with Delta strain showed no viable virus load while the control group exhibited more than 10^3^ PFU/g of tissue (Fig. 5d). Despite the absence of viable virus in immunized animals, viral RNA was found in lung samples at 5 DPI (*p* < 0.05) (Fig. 5e). For mice challenged with the Gamma variant, one immunized mouse died at 9 DPI and the protection observed was almost 90% (Fig. 5f, g). Significant reduction in viable virus load and viral RNA was observed in lung samples from immunized mice and challenged with Gamma variant (*p* < 0.01) (Fig. 5h, i). Knowing that infection with the Omicron variant resulted in a non-lethal infection, after challenge with Omicron, viral load was evaluated in nasal wash and lungs. Immunized mice were euthanized at 3 and 6 DPI and no change in weight and survival was observed (Fig. 5j, k). After challenge with Omicron, pCTV-WS immunized mice showed a significant reduction in viable virus load at 3 DPI in nasal wash and lung in comparison to control group (*p* < 0.01) (Fig. 5l, m). Viral RNA persistence, also evaluated in nasal wash and lung at 3 and 6 DPI showed a significant reduction in pCTV-WS immunized animals at both time points in comparison to control group (*p* < 0.01) (Fig. 5n, o). Histopathological analysis from non-immunized animals challenged with Omicron variant showed intense and diffuse inflammatory infiltrate (black arrows) in entire lung parenchyma associated with inflammatory perivascular infiltrate (red arrow), congestion (blue arrow) and hemorrhage (green arrow) (Fig. 5p). On the other hand, pCTV-WS immunized mice presented preserved lung parenchyma and intact alveolar spaces (Fig. 5q).

### pCTV-WS immunization also induces a strong humoral immune response and protective immunity in hamsters

**Fig. 6:**
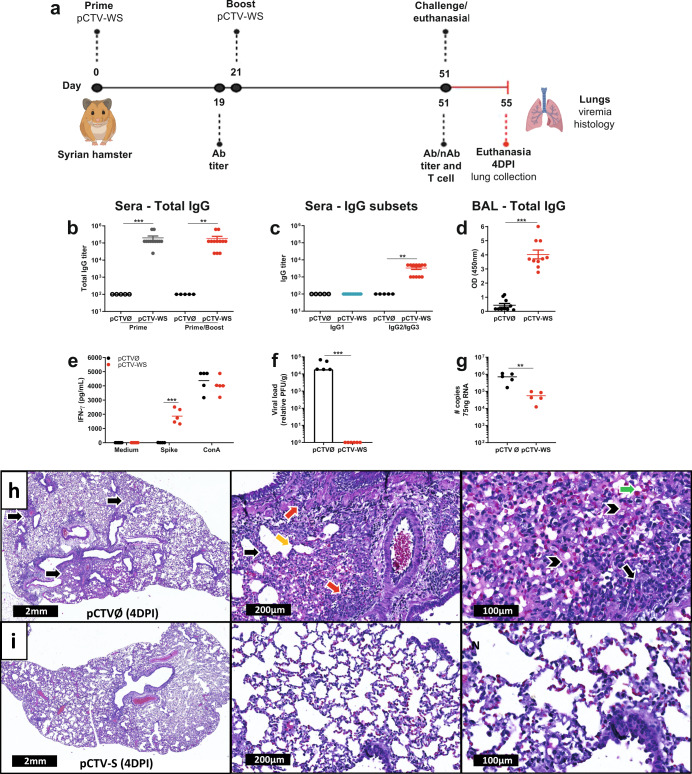
pCTV-WS immunogenicity and protective immunity in Syrian hamster model. **a** Schematic representation of the experimental design used to assess the humoral and cellular immune response and protection against SARS-CoV-2. **b** Anti-WS total IgG titer in pCTVØ (open circles) or pCTV-WS (gray circles) immunized hamsters after 1^st^ immunization (prime) and pCTVØ (filled circle) or pCTV-WS (red circles) after prime/boost [*n* = 5, pCTVØ and *n* = 12, pCTV-WS]. **c** IgG1 (blue circles) and IgG2/IgG3 (red circles) subclass 30 days after prime/boost pCTV-WS immunization. IgG1 (open circle) and IgG2/IgG3 (filled circle) in the control group [*n* = 5, pCTVØ and *n* = 12, pCTV-WS]. **d** Anti-WS total IgG in bronchoalveolar lavage (BAL) of pCTVØ (black circles) or pCTV-WS (red circles) immunized hamsters [*n* = 10 mice/group]. **e** IFN-γ levels secreted by splenocytes from pCTVØ (black circles) or pCTV-WS (red circles) immunized hamsters after 72 h of incubation with medium (negative control), restimulation with recombinant WS protein or Concanavalin A (positive control). Lung viral load **f** and SARS-CoV-2 RNA copy number **g** in pCTVØ (black circles) or pCTV-WS (red circles) immunized hamsters and challenged with the Wuhan strain at 4 DPI [*n* = 5 mice/group]. Histopathological sections of lungs from pCTVØ **h** or pCTV-WS **i** immunized hamsters at 4 DPI. Inflammatory infiltrate (black arrow), perivascular inflammatory infiltrate (red arrow), alveolar septal thickening (yellow arrow), edema (arrow head) and hemorrhage (green arrow) at 1.1×, 10×, and 20× magnification. The statistical analysis was performed using unpaired t-test or Mann-Whitney U test, according to data distribution. **p < 0.01; ***p < 0.001.

Hamsters were immunized with a two-dose i.m. of 100 μg of plasmid 21 days apart in order to assess the vaccine candidate immunogenicity (Fig. 6a). pCTV-WS induced high levels of serum IgG in hamsters both after prime and boost (Fig. 6b), and in BAL after prime/boost (*p* < 0.001) (Fig. 6d). Subclass IgG analysis showed that the immunization elicited both IgG2 and IgG3 to WS protein (Fig. 6c). Individual antibody curves for IgG subsets are presented in Supplementary Fig. 1g, h. Splenocytes isolated from hamsters 30 days after the pCTV-WS second dose and restimulated with 10 μg/mL of recombinant WS protein induced the secretion of high levels of IFN-γ (*p* < 0.001) (Fig. 6e).

Although the pCTV-WS immunized hamsters have a viral load below detection limit (Fig. 6f), viral RNA persisted in lung samples at 4DPI at a 10-fold lower levels compared to the control mice (*p* < 0.01) (Fig. 6g). Histopathological analysis from non-immunized animals showed several focuses of inflammation (black arrows) with a strong inflammation around vessels and bronchi (red arrow) and alveolar septum thickening (yellow arrow) (Fig. 6h). Lungs from these animals also showed hemorrhagic foci (green arrow) and edema (arrowhead) (Fig. 6h). On the other hand, pCTV-WS immunized hamsters presented much less inflammatory infiltrates compared to control animals and large areas of preserved lung parenchyma (Fig. 6i). Hamster normal lung tissue sections can be observed in Supplementary Fig. 2b.

### pCTV-WS immunization also induces protection against SARS-CoV-2 variants in moderate COVID-19 experimental model

**Fig. 7:**
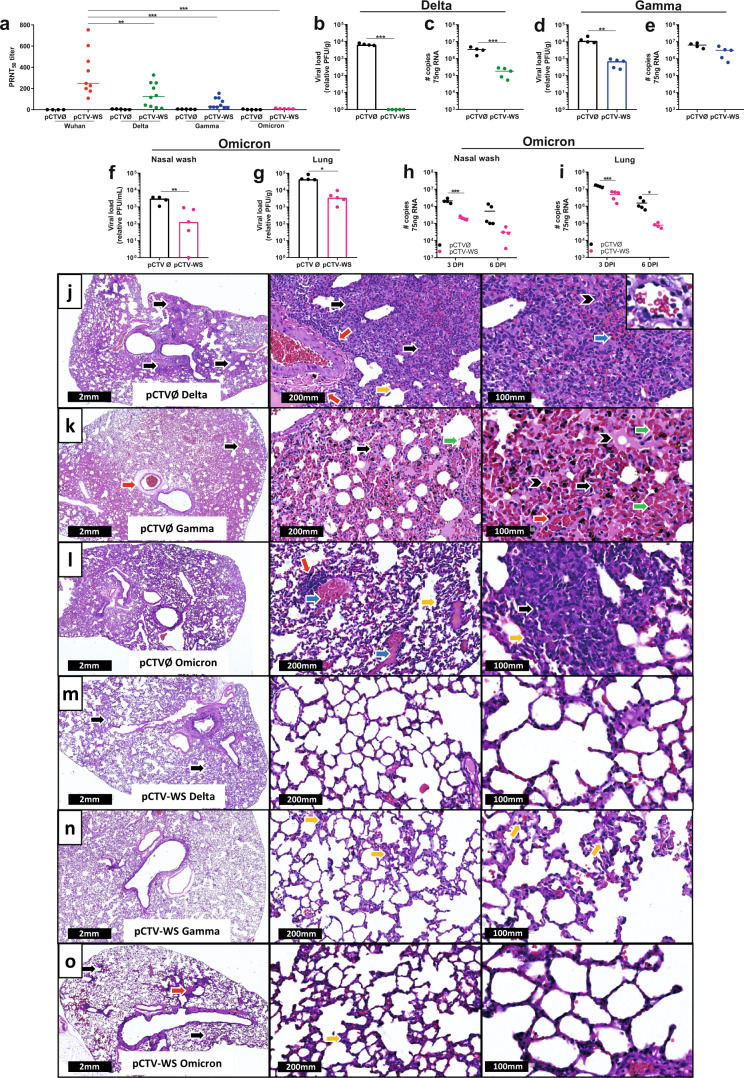
Protection against SARS-CoV-2 variants of concern in pCTV-WS immunized Syrian hamsters. **a** PRNT50 was evaluated using different SARS-CoV-2: Wuhan strain (B lineage), Delta, Gamma and Omicron variants in sera samples from pCTVØ (black circles) or pCTV-WS (colored lines) immunized hamsters [*n* = 4–5, pCTVØ and *n* = 4–10, pCTV-WS]. Viral load **b** and SARS-CoV-2 RNA copy number **c** in lung samples of pCTVØ (black circles) or pCTV-WS (green circles) immunized hamsters and challenged with the Delta variant of SARS-CoV-2 at 4 DPI [*n* = 4–5 hamsters/group]. Viral load **d** and SARS-CoV-2 RNA copy number **e** in lung samples of pCTVØ (black circles) or pCTV-WS (blue circles) immunized hamsters and challenged with the Gamma variant of SARS-CoV-2 at 4 DPI [*n* = 4–5 hamsters/group]. Viral load in nasal wash **f** and lung **g** of pCTVØ (black bar) or pCTV-WS (pink bar) immunized hamsters and challenged with the Omicron variant at 3 DPI [*n* = 4–5 hamsters/group]. SARS-CoV-2 RNA copy number in nasal wash **h** and lung tissue **i** of pCTVØ (black circles) or pCTV-WS (pink circles) immunized hamsters and challenged with the Omicron variant at 3 and 6 DPI [*n* = 4–5 hamsters/group]. Histopathological sections of lungs from pCTVØ **j**–**l**, or pCTV-WS **m**–**o** immunized hamsters and challenge with the Delta, Gamma and Omicron variants at 4 DPI, respectively. Inflammatory infiltrate (black arrow), perivascular inflammatory infiltrate (red arrow), alveolar septal thickening (yellow arrow), vascular congestion (blue arrow), edema (arrow head) and hemorrhage (green arrow) at 1.1×, 10×, and 20× magnification [*n* = 4-5 hamsters/group]. The statistical analysis was performed using unpaired t-test or Mann-Whitney U test, according to data distribution. *p < 0.05; **p < 0.01; ***p < 0.001.

Sera from all immunized hamsters showed positive results for neutralizing antibodies to the Wuhan SARS-CoV-2 (Fig. 7a). PRNT50 titers were highest against Delta and lowest against Omicron. A significant reduction in nAbs titer for all variants was observed (*p* < 0.01), being more pronounced against Omicron variant with no animal showing neutralization titer (Fig. 7a). The neutralizing curves with different dilutions of sera were presented in Supplementary Fig. 1i–l. Viral load was also assessed in lung samples at 3–4DPI for the three variants (Fig. 7b–i). After challenge with the Delta variant, the immunized animals showed no PFU (*p* < 0.001) and lower levels of viral RNA (*p* < 0.001) in lung samples when compared to the control group (Fig. 7b, c). pCTV-WS immunization was also able to reduce the number of PFUs in the lung from hamsters challenged with the Gamma variant in approximately 10-fold compared to the control group (*p* < 0.01) (Fig. 7d). No statistically significant difference was observed in lung for viral RNA levels at 4DPI (Fig. 7e). pCTV-WS immunized animals also showed reduced viral load in both nasal wash (Fig. 7f) and lung (Fig. 7g) at 3 DPI with the Omicron variant (*p* < 0.05). Lower levels of viral RNA were also detected at 3 DPI in the nasal wash of immunized animals (*p* < 0.001) (Fig. 7h). Reduced levels of viral RNA were detected at 3 and 6 DPI in lung tissue (*p* < 0.05) (Fig. 7i).

Histopathological analyses were performed with tissues from hamster that were challenged with the three different variants of SARS-CoV-2. Hamsters immunized with pCTVØ and challenged with either the Delta, Gamma and Omicron variants showed diffuse tissue inflammatory infiltrate (black arrow), perivascular inflammatory infiltrate (red arrow), alveolar septal thickening (yellow arrow), vascular congestion (blue arrow), and edema (arrow head) (Fig. 7j–l). Hemorrhage was observed only in mice challenged with Gamma variant (green arrow) (Fig. 7k). On the other hand, animals immunized with pCTV-WS had maintained tissue architecture and only discrete inflammatory infiltrate (black arrow), perivascular inflammatory infiltrate (red arrow) and alveolar septal thickening (yellow arrow) (Fig. 7m–o).

### Differential requirement of nAbs and T cells in protective immunity elicited by pCTV-WS

**Fig. 8:**
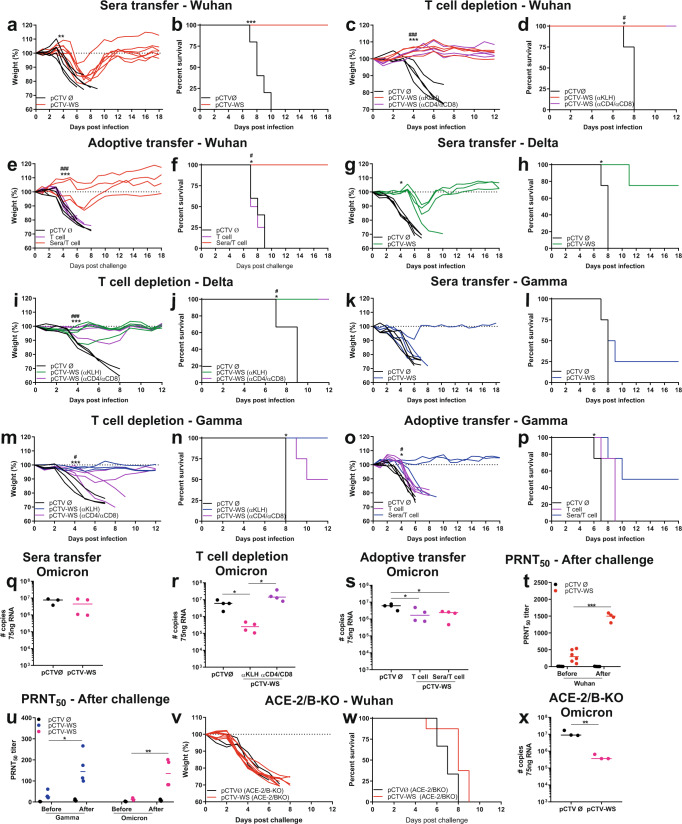
Neutralizing antibodies and protective immunity in pCTV-WS immunized K18-hACE2 mice. Body weight **a** and survival **b** of K18-hACE2 mice after sera transfer from pCTVØ (black lines) or pCTV-WS (red lines) immunized mice followed by challenge with the Wuhan strain [*n* = 5, pCTVØ and *n* = 7 pCTV-WS]. Body weight **c** and survival **d** of pCTVØ (black lines) and pCTV-WS immunized K18-hACE2 mice, treated with αKLH (red lines) or αCD4/αCD8 (purple lines) and challenge with the Wuhan strain [*n* = 4 mice/group]. Statistical analysis: [^*^pCTVØ *vs* pCTV-WS (αKLH)] [^#^pCTVØ *vs* pCTV-WS (αCD4/αCD8)]. Body weight **e** and survival **f** of K18-hACE2 mice (black lines), receiving adoptive transfer of T cells (purple lines) or adoptive transfer of T cell + sera transfer (red lines) followed by challenge with Wuhan strain [*n* = 4 mice/group]. Statistical analysis: [^*^pCTVØ *vs* sera + T cell] [^#^sera + T cell *vs* T cell]. Body weight **g** and survival **h** of K18-hACE2 mice (black lines), and receiving sera transfer (green lines) followed by challenge with Delta variant [*n* = 4 mice/group]. Body weight **i** and survival **j** of pCTVØ (black lines) and pCTV-WS immunized K18-hACE2 mice, treated with αKLH (green lines) or αCD4/αCD8 (purple lines) and challenged with Delta variant [*n* = 3–4 mice/group]. Statistical analysis: [^*^pCTVØ *vs* pCTV-WS (αKLH)] [^#^pCTVØ *vs* pCTV-WS (αCD4/αCD8)]. Body weight **k** and survival **l** of K18-hACE2 mice (black lines), and receiving sera transfer (blue lines) followed by challenge with Gamma variant [*n* = 4 mice/group]. Body weight **m** and survival **n** of pCTVØ (black lines) and pCTV-WS immunized K18-hACE2 mice, treated with αKLH (blue lines) or αCD4/αCD8 (purple lines) and challenged with Gamma variant [*n* = 3–4 mice/group]. Statistical analysis: [^*^pCTVØ *vs* pCTV-WS (αKLH)] [^#^pCTVØ *vs* pCTV-WS (αCD4/αCD8)]. Body weight **o** and survival **p** of K18-hACE2 mice (black lines), receiving adoptive transfer of T cells (purple lines) or adoptive transfer of T cell + sera transfer (blue lines) followed by challenge with Gamma variant [*n* = 4 mice/group]. Statistical analysis: [^*^pCTVØ *vs* sera + T cell] [^#^pCTVØ *vs* T cell]. **q** SARS-CoV-2 RNA copy number from K18-hACE2 that received sera transfer from pCTVØ (black circles) or pCTV-WS (pink circles) immunized mice and challenged with the Omicron variant at 6 DPI [*n* = 4 mice/group]. **r** SARS-CoV-2 RNA copy number from K18-hACE2 mice immunized with pCTVØ (black circles) and pCTV-WS that received isotype control antibody (αKLH - pink circles) or αCD4/αCD8 (purple circles) and challenged with the Omicron variant at 6 DPI [n = 4 mice/group]. **s** Viral RNA load of K18-hACE2 mice that received adoptive T cell transfer (purple circles) or adoptive T cell transfer + sera transfer (pink circles) and challenged with the Omicron variant at 6 DPI [*n* = 4 mice/group. PRNT50 of K18-hACE2 mice immunized with pCTVØ (black circles) or pCTV-WS (colored circles) before and after challenge with Wuhan strain **t** or with Gamma and Omicron variants **u** [*n* = 4–6 mice/group]. Body weight **v** and survival **w** of K18-hACE2/B-KO mice immunized with pCTVØ (black lines) or pCTV-WS (red lines) and challenged with the Wuhan strain [*n* = 3–9 mice/group]. **x** Viral RNA load of K18-hACE2/B-KO mice immunized with pCTVØ (black circles) or pCTV-WS (pink circles) and challenged with Omicron variant [*n* = 3 mice/group]. Data pooled from one or two independent experiments. The statistical analysis of weight curves was performed by area under curve followed by unpaired t-test or Mann-Whitney U test, according to the data distribution. The statistical analysis of survival was performed using the log-rank test. The statistical analysis for RNA copy number was performed using unpaired t-test or Mann-Whitney U test, according to data distribution. *p < 0.05; **p < 0.01; ***p < 0.001.

Sera samples collected from mice that received 2 doses of pCTVØ or pCTV-WS were transfered to recipient mice one day before challenge with Wuhan SARS-CoV-2. Initially, all mice lost weight, independently of the sera transferred. However, mice that received sera sample from a pCTV-WS donor recovered the initial body weight (*p* < 0.01) and survived up to 18 DPI whereas all the mice that received sera from a pCTVØ donor died up to 10 DPI (*p* < 0.001) (Fig. 8a, b). We also investigated whether T cells are essential for protective immunity against SARS-CoV-2. On days -3, -2, and -1 before challenge, pCTV-WS immunized K18-hACE2 mice were treated with anti-CD4 and anti-CD8 antibodies or KLH (isotype control). Depletion of T cell did not influence the course of infection and all mice challenged with the Wuhan strain maintained body weight (*p* < 0.001) and survived (*p* < 0.05) (Fig. 8c, d). Adoptive T cell transfer did not protect mice from infection and all animals died up to 9 DPI, indicating the importance of nAbs against Wuhan strain (Fig. 8e, f).

For Delta variant an elevated protection (75% survival) was found in mice that received sera from mice immunized with pCTV-WS (Fig. 8g, h). In addition, all pCTV-WS immunized mice that had T cells depleted survived (Fig. 8i, j). In contrast, only partial protection (25% survival) was observed for Gamma variant in K18-hACE2 mice that received sera from pCTV-WS immunized mice before challenge (Fig. 8k–l). Moreover, after challenge with the Gamma variant, survival dropped to 50% in immunized mice depleted of T cells (Fig. 8m, n). Adoptive T cell transfer did not protect mice from infection with Gamma variant and all mice died up to 9 DPI. Nevertheless, simultaneous transference of immune sera and T cells from vaccinated mice protected 50% of animals, demonstrating the importance of both humoral and cellular immune response against this variant (Fig. 8o, p).

For Omicron variant, we also analyzed the viral RNA load in naïve mice that received serum transfer and in pCTV-WS-immunized mice depleted of T cells. Mice that received sera transfer had similar viral RNA load when compared to non-immunized control group (Fig. 8q). On the other hand, T cell-depleted animals showed higher viral RNA load in comparison to immunized mice that received the isotype control (αKLH) and similar load to non-immunized animals (pCTVØ) (Fig. 8r). Adoptive transfer of T cells with or without sera transfer (*p* < 0.05) showed a similar reduction in viral RNA load in K18-hACE2 mice (Fig. 8s), suggesting the absence of nAbs against this variant. In addition, the PRNT50 was calculated before and after challenged. Sera from mice immunized and challenged with Wuhan strain showed a significant increase in neutralizing capacity (*p* < 0.001) (Fig. 8t). However, immunized mice, challenged with either Gamma and Omicron variants showed 10-fold less neutralizing capacity (*p* < 0.05) as compared to mice challenged with Wuhan (Fig. 8u), further questioning the efficacy of nAbs in protective immunity against the Gamma and Omicron variants.

We also analyzed body weight and survival of K18-hACE2/B-KO mice immunized with pCTV-WS and challenged with Wuhan strain or the Omicron variant. All mice died up to 9 DPI, confirming the importance of nAbs in protecting against the ancestral strain (Fig. 8v–w). On the other hand, K18-hACE2/B-KO mice immunized with pCTV-WS and challenged with Omicron variant showed reduction in viral RNA load (*p* < 0.01), similar to the vaccinated K18-hACE2 mice (Fig. 8x). All together, these results demonstrate that protective immunity against Wuhan strain (B lineage) and Delta variant is primarily mediated by nAbs, whereas protection in immunized mice against Gamma seems to be mediated by both nAbs and T cells. Importantly, protective immunity to Omicron variant is predominantly mediated by T cells.

## Discussion

Although vaccine coverage against SARS-CoV-2 is broad in most high- and middle-income countries, the COVID-19 pandemic continues with new waves of high transmission. The escape of emergent variants^23^ and the waning of vaccine-induced immunity^24,25^ contribute to the limited control of COVID-19 pandemic. Nevertheless, it seems that vaccination will remain essential for reducing severe cases and deaths. We developed a plasmid DNA vaccine containing the full-length S gene from the Wuhan strain (pCTV-WS), which might be useful as a booster vaccine in the context of the new variants. Preclinical studies using animal models have provided important data on pathogenicity, transmissibility, as well as escape from infection- or vaccine-induced immunity by VOCs^26^. Such information may be used to establish the protection level of vaccines produced against the ancestral strain and the immune mechanisms involved. The non-human primates (NHP) have been widely used to test COVID-19 vaccines against the SARS-CoV-2 variants. While NHP are good models for SARS-CoV-2 infection, they are not good models for severe COVID-19. Here, we evaluated the pCTV-WS efficacy in K18-hACE2 mice and hamsters, which are models of severe and moderate COVID-19, respectively. Our results show that while pCTV-WS-induced nAbs are critical to control infection with the ancestral Wuhan and the Delta variant, T cells had a critical role in controlling viral load and disease in immunized mice infected with either the Gamma or Omicron VOCs.

The Omicron variant has spread globally rapidly and is the variant with the most notable antibody escape to date^22,27^. Lower in vitro neutralization titers of sera from convalescent and vaccinated individuals are consistent with reduced effectiveness of vaccines in use against Omicron and other variants^28,29^. Although the variant can infect and cause disease in either vaccinated or convalescent individuals, innate immunity, pre-existing T cell response, non-neutralizing antibodies and residual nAbs appear to protect against severe disease^30,31^, reducing hospitalizations and deaths. While booster doses improve immunity, some studies also indicate that booster doses with vaccines produced in different platforms induce more robust immune responses against variants, though for short periods of time^32,33^.

To date, four vaccines based on S protein-encoding DNA against SARS-CoV-2 are ongoing with phase 3 clinical trials. Preliminary data reported from three of these vaccines have shown that they are efficacious, safe, well tolerated and induce both humoral and cellular immune responses^34–36^. Here, we show that two doses three weeks apart of pCTV-WS induced high levels of nAbs and robust T cell responses, both in mice and hamsters. Histopathological analysis of animals challenged with Wuhan strain and different variants showed that vaccinated mice and hamsters had preserved lung structure, reduced inflammatory infiltrate, pro-inflammatory cytokines and chemokines levels compatible with controlled viral replication, as compared to non-vaccinated animals. The neutralization assays with sera from mice or hamsters immunized with pCTV-WS showed cross-neutralization for the ancestral B lineage of SARS-CoV-2 and the Delta variant. However, it failed to induce high levels of nAbs to the highly virulent Gamma isolate. Consistent with the multiple mutations in the S protein, the Omicron variant completely escape the neutralizing nAbs induced by the DNA-WS, both in vitro and in vivo.

While waning antibody are observed in mRNA vaccinees, memory T and B cells remain relatively stable^37^. Although T cells cannot prevent infection, the immunity conferred by these cells is still essential to limit virus replication and spread to the lower respiratory tract and prevent severe disease^38^. Another study showed that T cell responses are largely preserved to Omicron Spike and non-Spike proteins in ~80% of previously infected and/or vaccinated individuals. Booster vaccination also increased T cell reactivity to Omicron Spike^39^. Thus, it is possible that protection against severe disease over 6 months among vaccinees is mediated by T cells. Importantly, mRNA, adenovirus and recombinant protein-based vaccines induce strong CD4^+^ and CD8^+^ T cell responses that recognize the VOCs, including the Omicron^40,41^.

The substantial Omicron variant/subvariants escape of neutralizing antibodies induced by both vaccination and previous infection^42^ at higher risk of vaccine breakthrough infections, has encouraged the production of bivalent COVID-19 vaccines based on the wild-type Spike protein combined with Omicron BA.1 or BA.4–5 Spike. Higher anti-Omicron BA.1 titers were observed with Omicron S-containing vaccines (mRNA-1273, Beta or Delta) compared to monovalent mRNA-1273 (Wuhan-1 SARS-CoV-2 strain) vaccine. However, the titers against Omicron BA.4/BA.5 were lower than against BA.1 for all candidate vaccines^43^. Another study has shown that a bivalent mRNA-1273 vaccine containing Omicron BA.1 Spike mRNA administered as a second boost induced higher antibody titers against Omicron BA.1 compared to monovalent mRNA-1273 vaccine^44^. In contrast, bivalent mRNA boosters encoding the Omicron BA.5^45^ or BA.4/BA.5^46^ and ancestral Spike showed similar anti-BA.5 or -BA.4/BA.5 nAb titers after boosting to the original monovalent and bivalent mRNA. Hence, while the capacity of the bivalent vaccines in inducing VOC-specific nAbs and improving protective immunity to SARS-CoV-2 variants are still under investigation, it is possible that their capacity to induce immune mediated resistance to Omicron subvariants remains largely dependent on effector T cells.

Importantly, studies performed in mice have shown that protection mediated by T-cells specific to SARS-CoV-2 can be achieved in the absence of nAbs^47–49^. A study identified SARS-CoV-2–specific T cell epitopes (several N, S, M, ORF peptides) recognized by CD4^+^ and CD8^+^ T cells in BALB/c and C57BL/6 mice. These T cells were polyfunctional and T cell vaccination alone partially protected SARS-CoV-2 infected mice from severe disease^47^. We have also evaluated a protein vaccine candidate constituted by the fusion of the Spike receptor binding domain (RDB) and the nucleocapsid (N) (SpiN chimera protein). Immunization with Poly ICLC adjuvanted SpiN induced robust virus-specific antibody and T cell responses and showed high efficacy in protecting against experimental challenge with SARS-CoV-2, even in absence of circulating nAbs at the time of infection. This vaccine also showed protection of rodents challenged with the ancestral Wuhan SARS-CoV-2 as well as the Delta and Omicron variants mediated by T-cell^48^. Protection against lethal infection with SARS-CoV-2 was also observed with three doses of a synthetic peptide vaccine in the absence of neutralizing antibodies. Repeated booster vaccinations suggested quantitatively and qualitatively improving CD8^+^ T cell response, leading to protection against lethal SARS-CoV-2 infection^49^. In addition, the depletion of T cells in a S protein vaccinated K18hACE2 mice and challenged with Beta variant resulted in higher viral titer in lung samples, in comparison to isotype antibody treated animals, showing the importance of T cells in the absence of nAbs^50^.

In fact, despite Omicron escape from nAbs induced by vaccine or previous infection, infected individuals have shown mild disease, indicating a possible involvement of T cells in disease modulation^51^. Here we performed a series of experiments using T cell depletion, adoptive sera and T cell transfers as well as immunized B cell-deficient K18-hACE2 mice. Our findings reinforced the hypothesis that T cells are critical for virus control during infection by the Omicron variant, since lower viral load was observed in immunized mice, even in absence of nAbs. In addition, for Gamma variant we observed requirement of both nAbs and T cells for optimal control of this VOC. An increase in the levels of circulating variant-specific nAbs was observed in mice vaccinated with pCTV-WS and challenged with either the Gamma or Omicron variants. However, the levels of variant-specific nAbs were 10-fold lower, when compared to the Wuhan-specific nAbs in pCTV-WS-vaccinated mice challenged with the ancestral SARS-CoV-2. Altogether, these findings are relevant for the development of the third-generation vaccines with broader efficacy against new variants and subvariants of SARS-CoV-2.

In conclusion, the rapid evolution and prevalence of the Omicron variant, followed by the subvariants, with greater potential for immune escape, have posed the challenge of updating the current vaccines with the S protein of emergent VOCs. However, vaccines employing ancestral SARS-CoV-2 targets are still important for protection to COVID-19, until we have more conclusive findings regarding vaccines adapted for variants. The plasmid DNA are low-cost vaccines and easy to be produced in industrial scale. In addition, they are highly stable, facilitating its distribution in countries with limited infra-structure for distribution of refrigerated and frozen vaccines. Added to that, the rapid adaptation to emergent/re-emergent pathogens suggest that plasmid DNAs are a promising platform to be used as boosters in transmission waves with new COVID-19 VOCs.

## Methods

### Study design

The aim of our study was to evaluate the immunogenicity and efficacy of a DNA-based COVID-19 vaccine against original and variants of SARS-CoV-2 in disease models (K18-hACE2 and hamsters). For this purpose, a plasmid containing full-length S gene sequence of the original SARS-CoV-2 strain was constructed (pCTV-WS). Expression of the cloned S gene sequence in pCTV vector was confirmed by Western blot and immunofluorescence techniques. We immunized K18-hACE2 transgenic mice, and hamsters with pCTV-WS and evaluated humoral and cellular immune responses by analyzing neutralizing and non-neutralizing antibodies levels, frequency of immune cells and cytokine and chemokine levels in lung tissue. We also used sera transfer, T cell depletion and adoptive T cell transfer techniques to evaluate the mechanisms of immune protection induced by the experimental pCTV-WS vaccine. Immunized animals were also infected with the original SARS-CoV-2 strain, and Delta, Gamma and Omicron variants at the biosafety level-3 lab to assess the level of protection by measuring body weight, survival, or viral load/viral RNA in nasal wash and lung. At the end of the experiments, the animals were euthanized for lung histopathological evaluation.

### Ethics statement and experimental models

All experiments with animals were carried out according to the principles of conduct of the Brazilian Guide of Practices for the Care and Use of Animals for Scientific and Didactic Purposes of CONSEA (http://www.sbcal.org.br/). Animal experimentation protocols were approved by the Committee on Ethics in the Use of Animals (CEUA) of Fundação Oswaldo Cruz (CEUA protocol LW25/20) and the Universidade de São Paulo (CEUA protocol 105/2020). Female WT C57BL/6 mice, 6–10 weeks old, were purchased from Center for Laboratory Animal Facilities of the Universidade Federal de Minas Gerais (CEBIO-UFMG). Human angiotensin-converting enzyme transgenic mice (K18-hACE2) in the C57BL/6 background, 6–10 weeks old, was originally obtained from Jackson Laboratories and were bred at Fiocruz-Minas Laboratory Animal Facilities. Human angiotensin-converting enzyme transgenic mice deficient in B cells (K18-hACE2/B-KO) in the C57BL/6 background, 6–10 weeks old, was bred at Faculdade de Medicina de Ribeirão Preto (USP). Golden Syrian Hamsters, 6–10 weeks old, were obtained at Fiocruz-Minas and used as a model of moderate COVID-19.

### Animal immunization and challenge experiments

C57BL/6, K18-hACE2 or K18-hACE2/B-KO mice and hamsters received two doses containing 100 μg of pCTVØ (sham-immunized) or pCTV-WS diluted in PBS, 21 days apart. The solution was administered intramuscularly in a final volume of 50 μl in each tibial muscle. Thirty days after the second dose, K18-hACE2 or K18-hACE2/B-KO mice were challenged intranasally with 5 × 10^4^ PFU of SARS-CoV-2 (for the Wuhan strain, Delta and Omicron variant) and 5 × 10^3^ PFU of SARS-CoV-2 (for the Gamma variant). Hamsters were challenged with 1 × 10^5^ PFU of SARS-CoV-2 (for the Wuhan strain, Delta and Omicron variant) and 1 × 10^4^ PFU of SARS-CoV-2 (for the Gamma variant). Body weight, clinical signs and survival were monitored daily after infection.

### CD4^+^/CD8^+^ T cell depletion, sera transfer and adoptive transfer

K18-hACE2 mice immunized with pCTV-WS were treated with 0.5 mg/mouse of rat anti-mouse CD4a mAb (clone GK1.5; Cat. # BE0003-1; BioXCell) and 0.5 mg/mouse of rat anti-mouse CD8a mAb (clone 2.43; Cat. # BE0061; BioXCell) or 0.2 mg/mouse of rat anti-KLH IgG (clone LTF-2; Cat. # BP0090; BioXCell). Antibodies were administered i.p. on days -3, -2 and -1 before infection and 7 days after infection. T cell depletion levels from treated animals were measured in blood samples by flow cytometry (Supplementary Fig. 3a). In the sera transfer assay, non-immunized K18-hACE2 mice, received 200 µL of sera i.p. from mice previously immunized with two doses of pCTV-WS one day before the infection. Transferred antibodies levels were determined using ELISA assay one day after sera transfer (Supplementary Fig. 3b). For T cell adoptive transfer, twelve animals immunized with pCTV-WS were euthanized 30 days after boosting. Spleen samples were collected to obtain splenocytes. T cells were purified using a Pan T cell isolation kit (Miltenyi Biotech) and 5 × 10^6^ purified T cells were adoptively transfer by i.v. injection into non-immunized K18-hACE2 mice one day before the infection. Purification percentage was determined by flow cytometry (Supplementary Fig. 3c).

### Viruses and cells

In this study, the SARS-CoV-2 WT strain (isolate BRA/SP02/2020) and Delta (EPI_ISL_2965577), Gamma (EPI_ISL_2499748) and Omicron (EPI_ISL_7699344) variants were used, isolated from clinical samples of Brazilian patients. Viruses were used to infect Vero E6 cells (ATCC CRL-1586) grown in Dulbecco’s Modified Eagle’s Medium (DMEM) (Gibco) supplemented with 1% penicillin/streptomycin (Sigma) and 2% fetal bovine serum (Sigma). After 2 days of infection, the culture supernatant was collected and clarified by centrifugation. The viral stocks obtained were titrated by the plaque-forming unit (PFU) method and stored at −80 °C.

### Plasmid construction, growth and purification

The pCTV plasmid containing the gene sequence encoding S protein, used in the immunization of experimental animals, was developed at the Centro de Tecnologias de Vacinas (UFMG). The S gene sequence of Wuhan SARS-CoV-2 codon-optimized was obtained from GenScript, cloned into pCTV and transformed in *E. coli* DH5α competent cells (Invitrogen) using the heat shock method. A positive colony was selected and grown in LB medium containing ampicillin (100 μg/mL) at 37 °C for 16 h. Finally, the bacteria were centrifuged and the plasmid (pCTV-WS) was purified using QIAGEN Plasmid Plus Giga Kit, according to manufacturer’s instructions.

### Western blot

HEK-293 cells (ATCC CRL-1573) were transfected with 10 μg of pCTV-WS plasmid using Fugene® transfection reagent (Promega). After 72 h of transfection the cells were collected and centrifuged. Pellets were resuspended in RIPA Buffer (Sigma) containing protease and phosphatase inhibitor cocktail (cOmplete ULTRA and PhosSTOP, Roche). Protein separation was performed on a 10% SDS-PAGE gel and transferred to a nitrocellulose membrane. Membranes were blocked with 3% bovine serum albumin (BSA) and incubated with anti-RBD primary antibody (Cat. # 40592-T62; 1:1000; Sino Biological) or anti-β-actin (Cat. # ab115777; 1:1000; Abcam). Next, membranes were incubated with secondary anti-rabbit antibodies (Cat. # A9169; 1:25,000; Sigma) conjugated with HRP and revealed using Clarity Max Western ECL Substrate (Bio-Rad). Images were captured with an Amersham Imager 600 (GE). Data were analyzed using ImageJ software (NIH). Blots samples derive from the same experiment and they were processed in parallel. Uncropped western blot membranes are shown in Supplementary Fig. 4a, b.

### Immunofluorescence

A 4-well chamber slide (Lab-Tek^®^II) was coated with Vero E6 cells (ATCC CRL-1586) and transfected with 5 µg pCTV-WS using Fugene® transfection reagent (Promega). The cells were fixed with formaldehyde 4% and permeabilized with PBS + 0.3% Triton X-100 (Sigma). Then, the cells were blocked with 1% BSA and incubated with sera sample from mice immunized with pCTVØ or pCTV-WS at 1:200 (v/v) dilution. Next, the cells were incubated with goat anti-mouse IgG Alexa Fluor 594 (Cat. # A-11032; 1:2000; Thermo Fisher). The slides were analyzed in the confocal microscope (Nikon). Images were processed with the NIS-Elements Viewer software (Nikon).

### Detection of hamster and mouse antigen-specific antibodies

Plates were coated overnight with 4 µg/mL of recombinant WS protein in carbonate buffer and blocked for 2 h with PBS containing 2% bovine serum albumin (PBS-BSA 2%) at 37 °C. Sera samples were serially diluted in PBS-BSA 2% and bronchoalveolar lavage (BAL) samples without any dilution were applied. After incubation for 1 h at 37 °C, the plates were washed and then incubated with total anti-mouse IgG (Cat. # 1030-05), anti-IgG1 (Cat. # 1070-05), anti-IgG2c (Cat. # 1079-05) or total anti-hamster IgG (Cat. # 6060-05), anti-IgG1 (Cat. # 1940-05), anti-IgG2/IgG3 (Cat. # 1935-05) conjugated with streptavidin-HRP (1:5000; Southern Biotech). After a few washes, the reaction was revealed using One-Step TMB (Scienco) for 20 min in the dark, and the reaction was stopped using 1 M H_2_SO_4_ (Sigma). Plates were read on Multiscan GO (Thermo Scientific) at 450 nm.

### Plaque Reduction Neutralization Test (PRNT) and Viral Load Test

Vero E6 cells were cultured in Dulbecco’s Modified Eagle’s Medium (DMEM) supplemented with 1% penicillin/streptomycin and 10% fetal bovine serum in 48-well plates. Mouse and hamster sera samples were inactivated at 56 °C and serially diluted in DMEM [1:10 to 1:320 (v/v)], mixed with 100 PFU each of SARS-CoV-2 viral stocks and incubated at 37 °C to determine the viral neutralizing capacity by sera antibodies from immunized animals. To perform the viral load assay, the right lung lobe was macerated in PBS (mice: 200 mg tissue/mL, hamster: 300 mg tissue/mL) using TissueLyser LT (Qiagen). Subsequently, the macerate was serially diluted in DMEM (undiluted to 1:100,000). For both techniques, the supernatant of Vero E6 cells was removed and the cells were inoculated with 50 μl/well of sera-virus mixture and incubated for 1 h at room temperature under gentle shaking, allowing the infection of cells by non-neutralized viral particles. Then, pre-warmed DMEM supplemented with 2% FBS and 2% carboxymethylcellulose (CMC) was gently added to the plates and incubated for 4 days at 37 °C and 5% CO_2_ to allow the viral plaque formation. Cells were fixed with 4% formaldehyde for 2 h and stained with a 1% solution of Naphtol blue black (Sigma) for 1 h for visualization of the plaques. The neutralizing activity was determined by plaques numbers reduction when compared to the positive control. The viral load was determined by counting the formed plaques.

### IFNγ measurement by Elisa and flow cytometry

Thirty days after administration of the second dose, the animals were euthanized and splenocytes were isolated by spleen maceration using a 100 μm pore cell strainer (Cell Strainer, BD Falcon). Then, erythrocytes were lysed with ammonium-chloride-potassium (ACK) buffer and the cell number was adjusted to 1 × 10^6^ cells/well and stimulated with 10 μg/mL of WS protein. As positive control, Concanavalin A at 5 μg/mL (Sigma) was used. The samples were then incubated for 72 h at 37 °C in 5% CO_2_. The culture supernatant was collected for determination of IFNγ levels by ELISA (mice: R&D Systems, hamster: Mabtech) following manufacturer’s instructions.

Splenocytes from immunized mice or challenged mice were isolated as described above and platted at 2 × 10^6^ cells/well for intracellular staining of IFN-γ. Splenocytes from immunized mice were stimulated with 10 μg/mL of WS protein for 24 h and in the last 4 h it was added PMA (50 ng/mL), Ionomycin (500 ng/uL), Golgi Plug (BD Bioscience) and Stop Golgi (BD Bioscience). Splenocytes from challenged mice were stimulated only with PMA and Ionomycin for 4 h in the presence of Golgi Plug and Stop Golgi. Cells were stained with Live/Dead stain (Acqua, Invitrogen) for 20 min at 4 °C in the dark, incubated with FcBlock (BD Bioscience) for 20 min at 4 °C and then stained with anti-CD3 (eFluor405; Cat. # 48-0032-82; 1:100; eBioscience), anti-CD4 (Fitc; Cat. # 553729; 1:250; BD Bioscience), anti-CD8 (APC-Cy7; Cat. # 100714; 1:200; Biolegend). Cells were permeabilized with Cytofix/Cytoperm (BD Bioscience) for 20 min at 4 °C in the dark and incubated with anti-IFN-γ (PerCP-Cy5.5; Cat. # 45-7311-82; 1:80; eBioscience) for 30 min at 4 °C. Representative density plots are presented in Supplementary Fig. 5 and the reference of antibodies used in Supplementary Table 2. Flow cytometry acquisition was performed using a BD LSRFortessa and ~200,000 live cells were acquired. Data were analyzed using FlowJo v10.5.3 software.

### Flow cytometry of lung tissue cells

Mice lungs were perfused with cold PBS, collected, digested with 100 μg/mL liberase (Roche) and incubated at 37 °C for 30 min for cell dissociation. Cells were filtered using a 100 μm pore cell strainer (Cell Strainer, BD Falcon) and purified using a 30% Percoll gradient. Erythrocytes were lysed with ammonium-chloride-potassium (ACK) buffer and the cell number was adjusted to 2 × 10^6^ cells/well. Cells were stained with Live/Dead stain (Acqua, Invitrogen) for 20 min at 4 °C in the dark, incubated with FcBlock (BD Bioscience) for 20 min at 4 °C and then stained with anti-CD3 (eFluor405; Cat. # 48-0032-82; 1:100; eBioscience), anti-CD4 (APC; Cat. # 100516; 1:200; Biolegend), anti-CD8 (APC-Cy7; Cat. # 100714; 1:200; Biolegend), anti-CD19 (BV570; Cat. # 115535; 1:160; Biolegend), anti-CD11b (PE-Cy7; Cat. # 25-0112-82; 1:4000; eBioscience), CD11c (AF700; Cat. # 56-0114-82; 1:100; eBioscience), DC-Sign (eFluor660; Cat. # 50-2094-82; 1:800; eBioscience), F4/80 (PE-Cy5; Cat. # 15-4801-82; 1:400; eBioscience), Ly6C (eFluor405; Cat. # 48-5932-82; 1:2000; eBioscience), Ly6G (FITC; Cat. # 11-9668-80; 1:250; eBioscience) and MHC II (PE; Cat. # 12-5320-82; 1:400; BD Bioscience). Gate strategy are presented in Supplementary Fig. 6 and the reference of antibodies used in Supplementary Table 2. Flow cytometry acquisition was performed using a BD LSRFortessa and ~200,000 live cells were acquired. Data were analyzed using FlowJo v10.5.3 software.

### Cytokines and chemokines measurements and viral load by qRT-PCR

For RNA isolation, a fragment of the left lung lobe was resuspended in Trizol Reagent (Invitrogen) and stored at −80 °C until RNA extraction, following manufacturer’s instructions. After total RNA extraction, samples were treated with DNase (Promega) and converted into cDNA using High-Capacity cDNA Reverse Transcription Kit (Applied Biosystems) according to manufacturer’s instructions. For cytokine and chemokines measurements, Sybr Green PCR Master Mix (Applied Biosystems) was used to carry out the reactions in an QuantStudio 12 K Flex Real-Time PCR System (Thermo Fisher Scientific) under standard conditions. qRT-PCR data were presented as 2^-ΔCt^. The primer sequences are presented in Supplementary Table 1. qRT-PCR was performed using the GoTaq Probe 1-step RT-qPCR System (Promega) for viral load quantification, according to the manufacturer’s instructions. Standard curves using serial dilutions of a plasmid containing the SARS-CoV-2 E gene sequence were used for sample quantifications. Reactions were carried out in QuantiStudio 5 Real-Time PCR System. Probes and primers sequences are also presented in Supplementary Table 1.

### Histopathology and immunohistochemical staining (IHC)

For histopathological analyses, lung fragments were fixed in 10% formaldehyde for seven days after collection. Samples were processed in alcohol and xylol using the PT05 TS tissue processor (LUPETEC, UK) and embedded in histological paraffin (Histosec®, Sigma). The 4 μm thick sections of tissues were performed using the microtome RM2125 RTS (Leica) and stained with hematoxylin and eosin.

Prior to initiating the IHC staining protocol, paraffin-embedded section tissue was deparaffinized and heat-mediated antigen retrieval with Tris/EDTA buffer pH = 9.0 was performed for 20 min at 60 °C. The endogenous peroxidase activity was blocked with 3% hydrogen peroxide for 30 min. Slides were incubated with anti-mouse CD4 (Cat. # ab183685; 1:50; Abcam), anti-mouse CD8 (Cat. # ab217344; 1:200; Abcam) and anti-mouse CD19 (Cat. # ab245235; 1:200; Abcam) primary antibodies overnight at 4 °C. Next, the slides were incubated with MACH 1 Universal HRP-Polymer (Biocare Medical, USA) for 30 min at RT, according to the manufacturer’s recommendations. Finally, the slides were stained using 3,3′-diaminobenzidine (DAB) chromogen (Biocare Medical, USA) and counterstained with Mayer’s hematoxylin. The histopathological and immunohistochemical analyses were carried out by two independent pathologists.

### Statistical analysis

Statistical analysis was performed using Prism software 7.0 for Windows (GraphPad Inc, USA). To remove possible outliers, the Grubb’s test was applied and the data distribution was verified using the Kolmogorov-Smirnoff test. Comparison between non-immunized and immunized groups was performed using unpaired t-test or Mann–Whitney U test, according to the data distribution. Multiple comparison analyses were performed using two-way ANOVA followed by Bonferroni post-hoc test. For survival analysis, the log-rank test was used. Statistical differences were considered significant when *p* values ≤ 0.05.

### Reporting summary

Further information on research design is available in the Nature Research Reporting Summary linked to this article.

## Supplementary information

Supplementary Files

REPORTING SUMMARY

## Data Availability

All data are available in the main text or the supplementary materials.

## References

[1] Centers for Disease Control and Prevention. Science brief: omicron (B. 1.1. 529) variant. Dec 2021. 34932278

[2] Phillips N (2021). The coronavirus is here to stay-here’s what that means. Nature.

[3] Callaway E (2020). The race for coronavirus vaccines: a graphical guide. Nature.

[4] Hassine H (2021). Covid‐19 vaccines and variants of concern: a review. Rev. Med. Virol..

[5] Mendonça SA, Lorincz R, Boucher P, Curiel DT (2021). Adenoviral vector vaccine platforms in the SARS-CoV-2 pandemic. npj Vaccines.

[6] Smith TRF (2020). Immunogenicity of a DNA vaccine candidate for COVID-19. Nat. Commun..

[7] Chavda VP, Pandya R, Apostolopoulos V (2021). DNA vaccines for SARS-CoV-2: toward third-generation vaccination era. Expert Rev. Vaccines.

[8] Kyriakidis NC, López-Cortés A, González EV, Grimaldos AB, Prado EO (2021). SARS-CoV-2 vaccines strategies: a comprehensive review of phase 3 candidates. npj Vaccines.

[9] Moderbacher CR (2020). Antigen-specific adaptive immunity to SARS-CoV-2 in acute COVID-19 and associations with age and disease severity. Cell.

[10] Sette A, Crotty S (2021). Adaptive immunity to SARS-CoV-2 and COVID-19. Cell.

[11] Sekine T (2020). Robust T cell immunity in convalescent individuals with asymptomatic or mild COVID-19. Cell.

[12] Grifoni A (2020). Targets of T cell responses to SARS-CoV-2 coronavirus in humans with COVID-19 disease and unexposed individuals. Cell.

[13] Riou R (2022). Escape from recognition of SARS-CoV-2 Beta variant spike epitopes but overall preservation of T cell immunity. Sci. Transl. Med..

[14] Jordan SC (2021). T cell immune responses to SARS-CoV-2 and variants of concern (Alpha and Delta) in infected and vaccinated individuals. Cell. Mol. Immunol..

[15] Zhou R (2022). Vaccine-breakthrough infection by the SARS-CoV-2 Omicron variant elicits broadly cross-reactive immune responses. Clin. Transl. Med..

[16] Ahmed SF, Quadeer AA, McKay MR (2022). SARS-CoV-2 T cell responses elicited by COVID-19 vaccines or infection are expected to remain robust against omicron. Viruses.

[17] Keeton R (2022). T cell response to SARS-CoV-2 spike T cross-recognize Omicron. Nature.

[18] GeurtsvanKessel CH (2022). Divergent SARS-CoV-2 Omicron–reactive T and B cell responses in COVID-19 vaccine recipients. Sci. Immunol..

[19] Gao Y (2022). Ancestral SARS-CoV-2-specific T cells cross-recognize the Omicron variant. Nat. Med..

[20] Goel RR (2021). mRNA vaccines induce durable immune memory to SARS-CoV-2 and variants of concern. Science.

[21] Geers D (2021). SARS-CoV-2 variants of concern partially escape humoral but not T-cell responses in COVID-19 convalescent donors and vaccines. Sci. Immunol..

[22] Liu L (2022). Striking antibody evasion manifested by the Omicron variant of SARS-CoV-2. Nature.

[23] Willett BJ (2022). SARS-CoV-2 Omicron is an immune escape variant with an altered cell entry pathway. Nat. Microbiol..

[24] Feikin DR (2022). Duration of effectiveness of vaccines against SARS-CoV-2 infection and COVID-19 disease: results of a systematic review and meta-regression. Lancet.

[25] Menni C (2022). COVID-19 vaccine waning and effectiveness and side-effects of boosters: a prospective community study from the ZOE COVID Study. Lancet Infect. Dis..

[26] Muñoz-Fontela C (2022). Advances and gaps in SARS-CoV-2 infection models. PLoS Pathog..

[27] Planas D (2022). Considerable escape of SARS-CoV-2 Omicron to antibody neutralization. Nature.

[28] Zou J (2022). Neutralization against Omicron SARS-CoV-2 from previous non-Omicron infection. Nat. Commun..

[29] Wang Q (2022). Antibody evasion by SARS-CoV-2 Omicron subvariants BA.2.12.1, BA.4, & BA.5. Nature.

[30] Flemming A (2022). Omicron, the great escape artist. Nat. Rev. Immunol..

[31] Bartsch YC (2022). Omicron variant Spike-specific antibody binding and Fc activity are preserved in recipients of mRNA or inactivated COVID-19 vaccines. Sci. Transl. Med..

[32] Clemens SAC (2022). Heterologous versus homologous COVID-19 booster vaccination in previous recipients of two doses of CoronaVac COVID-19 vaccine in Brazil (RHH-001): a phase 4, non-inferiority, single blind, randomised study. Lancet.

[33] Zuo F (2022). Heterologous immunization with inactivated vaccine followed by mRNA-booster elicits strong immunity against SARS-CoV-2 Omicron variant. Nat. Commun..

[34] Tebas P (2021). Safety and immunogenicity of INO-4800 DNA vaccine against SARS-CoV-2: a preliminary report of an open-label. Phase 1 Clin. trial EClinicalMedicine.

[35] Khobragade A (2022). Efficacy, safety, and immunogenicity of the DNA SARS-CoV-2 vaccine (ZyCoV-D): the interim efficacy results of a phase 3, randomised, double-blind, placebo-controlled study in India. Lancet.

[36] Ahn JY (2022). Safety and immunogenicity of two recombinant DNA COVID-19 vaccines containing the coding regions of the spike or spike and nucleocapsid proteins: an interim analysis of two open-label, non-randomised, phase 1 trials in healthy adults. Lancet Microbe.

[37] Zhang Z (2022). Humoral and cellular immune memory to four COVID-19 vaccines. Cell.

[38] Wherry EJ, Barouch DH (2022). T cell immunity to COVID-19 vaccines. Science.

[39] Naranbhai V (2022). T cell reactivity to the SARS-CoV-2 Omicron variant is preserved in most but not all individuals. Cell.

[40] Tarke A (2022). SARS-CoV-2 vaccination induces immunological T cell memory able to cross-recognize variants from Alpha to Omicron. Cell.

[41] Heitmann JS (2022). A COVID-19 peptide vaccine for the induction of SARS-CoV-2 T cell immunity. Nature.

[42] Cao Y (2022). BA. 2.12. 1, BA. 4 and BA. 5 escape antibodies elicited by Omicron infection. Nature.

[43] Branche, A. R. et al. SARS-CoV-2 Variant Vaccine Boosters Trial: Preliminary Analyses. Preprint at https://www.medrxiv.org/content/10.1101/2022.07.12.22277336v1 (2022).

[44] Chalkias S (2022). A bivalent Omicron-containing booster vaccine against COVID-19. N. Engl. J. Med..

[45] Collier AR (2023). Immunogenicity of BA.5 bivalent mRNA vaccine boosters. N. Eng J. Med.

[46] Wang Q (2022). Antibody evasion by SARS-CoV-2 Omicron subvariants BA. 2.12. 1, BA. 4 and BA. 5. Nature.

[47] Zhuang Z (2021). Mapping and role of T cell response in SARS-CoV-2-infected mice. J. Exp. Med..

[48] Castro JT (2022). Promotion of neutralizing antibody-independent immunity to wild-type and SARS-CoV-2 variants of concern using an RBD-Nucleocapsid fusion protein. Nat. Commun..

[49] Pardieck IN (2022). A third vaccination with a single T cell epitope confers protection in a murine model of SARS-CoV-2 infection. Nat. Commun..

[50] Kingstad-Bakke B (2022). Vaccine-induced systemic and mucosal T cell immunity to SARS-CoV-2 viral variants. Proc. Natl Acad. Sci. USA.

[51] Goldblatt D (2022). Correlates of protection against SARS‐CoV‐2 infection and COVID‐19 disease. Immunol. Rev..

