# Supplementary material for: Differential requirement of neutralizing antibodies and T cells on protective immunity to SARS-CoV-2 variants of concern
Source: NPJ Vaccines. 2023 Feb 13;8:15. doi: 10.1038/s41541-023-00616-y (PMC9923671; doi:10.1038/s41541-023-00616-y)
Supplement: Supplementary file 1 — Supplementary Files [file 41541_2023_616_MOESM1_ESM.pdf]

SUPPLEMENTARY FILES

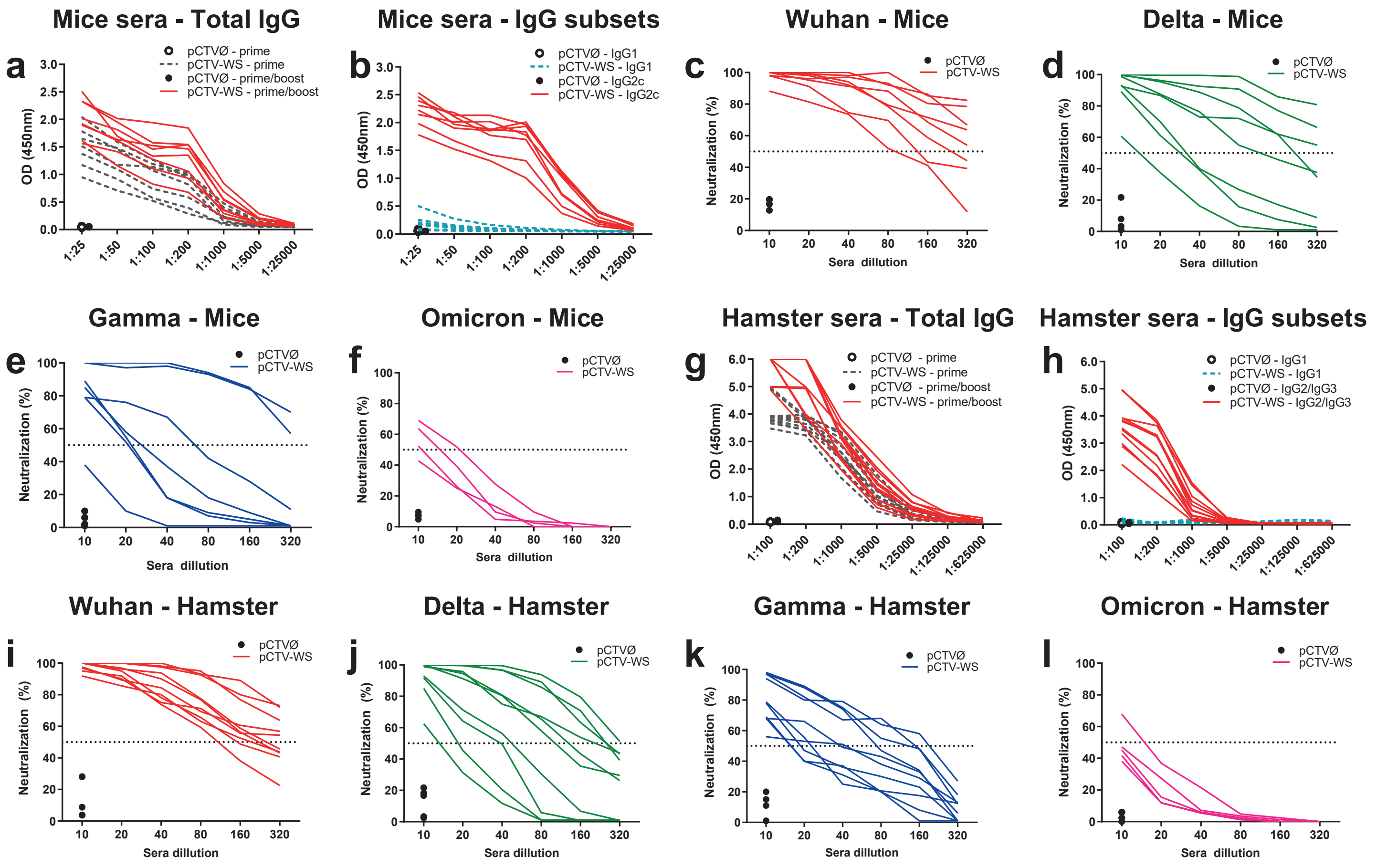

Supplementary Figure 1: Individual antibody and nAbs curves from immunized mice and hamsters. (a) Antibody curves of sera samples from mice immunized after first dose with pCTVØ (open circles) or pCTV-WS (gray dotted lines) and 30 days after the second dose (pCTVØ: filled circles and pCTV-WS: red lines). **(b)** IgG1 (blue dotted lines) and IgG2c (red lines) antibody curves of sera samples from mice immunized with pCTV-WS or pCTVØ (open and filled circles) [n = 8 mice/group]. Neutralization percentage was evaluated using different SARS-CoV-2: Wuhan strain (B lineage) **(c)**, Delta **(d)**, Gamma **(e)** and Omicron **(f)** variants in sera samples from pCTVØ (black circles) or pCTV-WS immunized K18-hACE2 mice (colored lines) [n = 4, pCTVØ and n = 4-8, pCTV-WS]. **(g)** Antibody curves of sera samples from hamster immunized after first dose with pCTVØ (open circles) or pCTV-WS (gray dotted lines) and 30 days after the second dose (pCTVØ: filled circles and pCTV-WS: red lines). **(h)** IgG1 (blue dotted lines) and IgG2/IgG3 (red lines) antibody curves of sera samples from hamster immunized with pCTV-WS or pCTVØ (open and filled circles) [n = 5, pCTVØ and n = 12, pCTV-WS]. Neutralization percentage was evaluated using different SARS-CoV-2: Wuhan strain (B lineage) **(i)**, Delta **(j)**, Gamma **(k)** and Omicron **(l)** variants in sera samples from pCTVØ (black circles) or pCTV-WS (colored lines) immunized hamsters [n = 4-5, pCTVØ and n = 4-10, pCTV-WS]. The statistical analysis was performed using unpaired t-test or Mann-Whitney U test, according to data distribution. $^{**}p<0.01$; $^{***}p<0.001$.

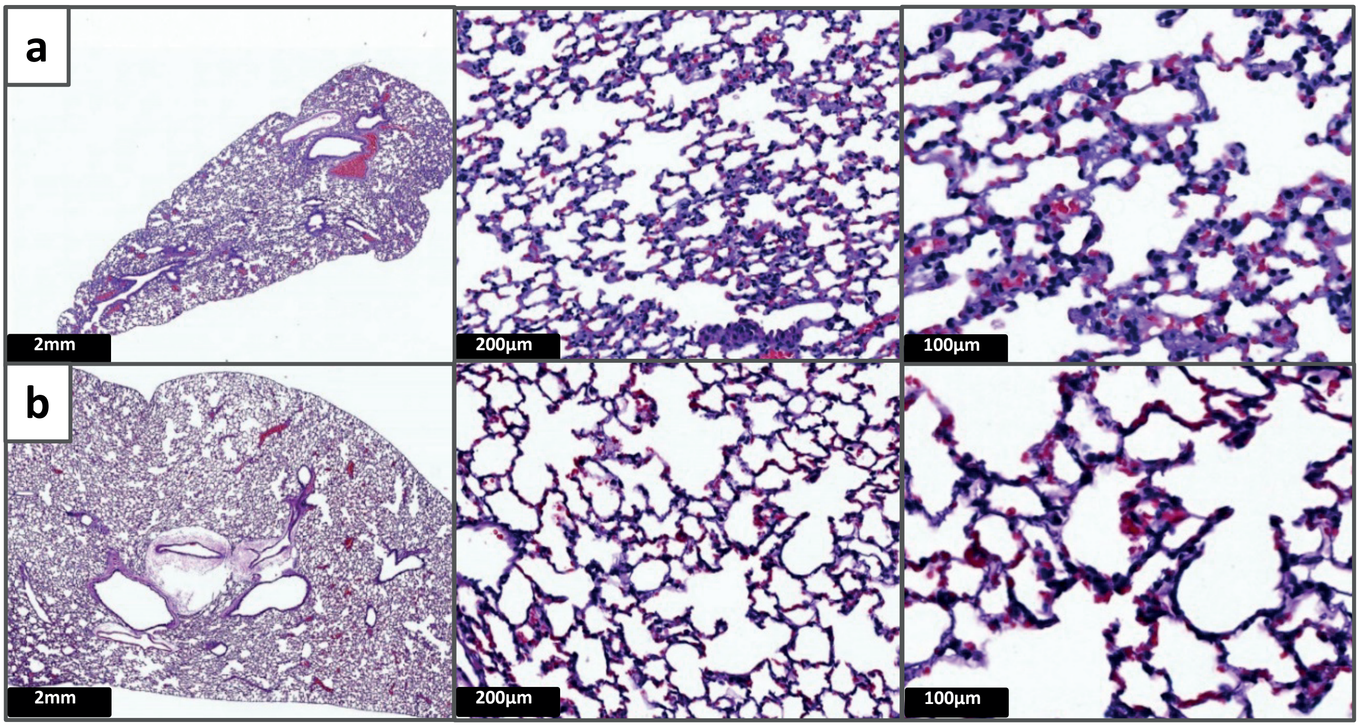

Supplementary Figure 2: Histology of lungs tissue sections from control mice and hamsters. Lung tissue sections from non-immunized and unchallenged mice **(a)** and hamsters **(b)** showing normal lung architecture at 1.1X, 10X and 20X magnification.

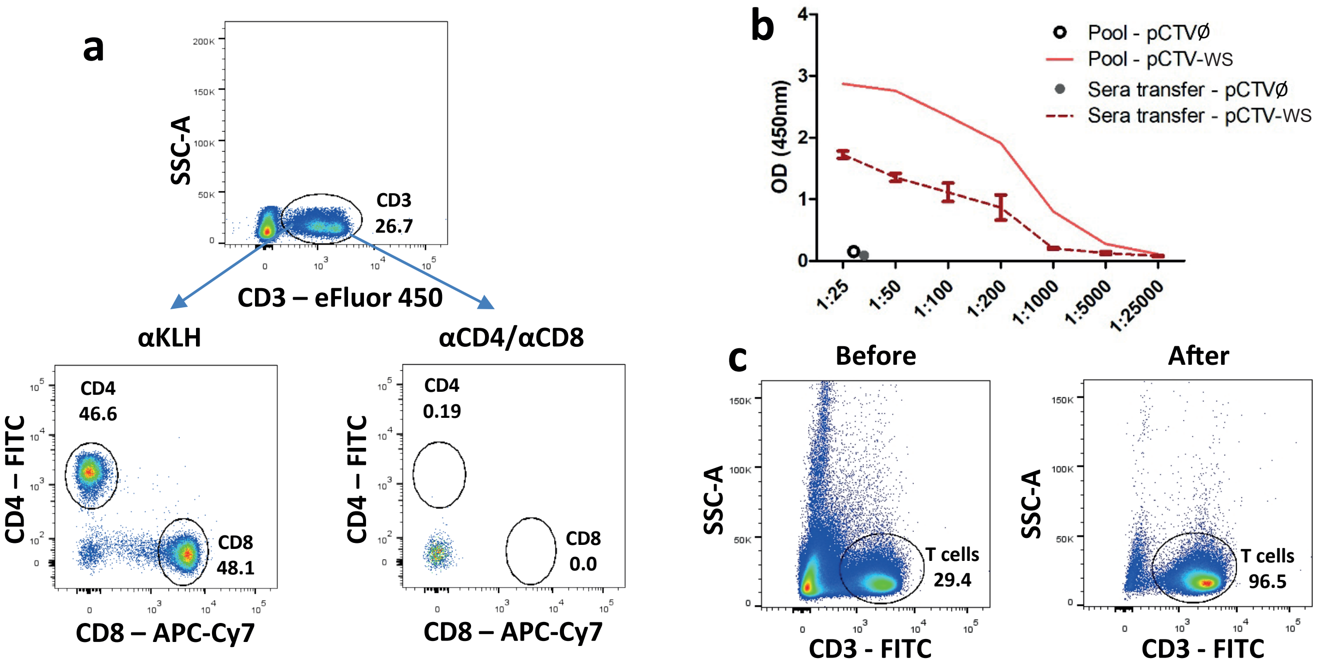

Supplementary Figure 3: Density plots representative of T cell depletion, total IgG titer in donor and recipient mice after sera transfer and T cell purification. (a) Representative density plots showing the frequency of CD4$^+$ and CD8$^+$ T cell subsets from K18-hACE2 mice treated with αKLH-isotype control (left panel) and depleted with αCD4$^+$/αCD8$^+$ antibodies (right panel). **(b)** Anti-WS total IgG titer of pooled sera sample from pCTVØ (open circles) or pCTV-WS (red line) immunized mice (prime/boost). Anti-WS total IgG titer in pCTVØ (gray circle) or pCTV-WS (dark red dotted line) recipient mice one day after sera transfer [n = 12 mice/pool and n = 2 mice/sera transfer]. **(c)** Representative density plots showing T cell (CD3$^+$) frequency before (left panel) and after enrichment (right panel) with magnetic beads.

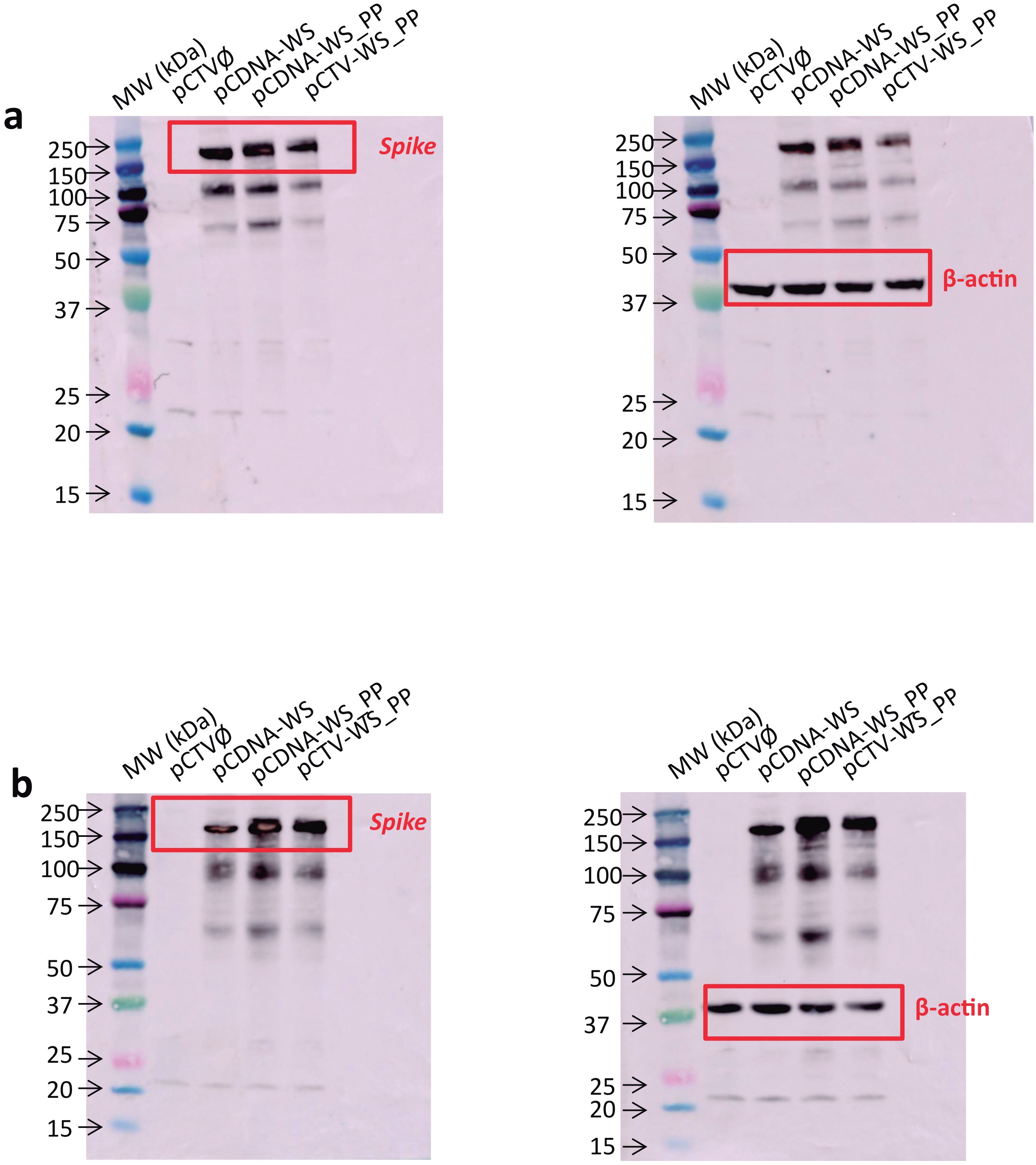

Supplementary Figure 4: Uncropped Western Blot membranes. (a-b) Western blot membranes showing the expression of WS protein in HEK293 cells transfected with the empty vector (pCTVØ), a commercial plasmid containing the full-length WS gene sequence of original SARS-CoV-2 isolated at Wuhan (pcDNA-WS), a commercial plasmid containing the codon-optimized and mutated sequence of SARS-COV-2 full-length S gene (pcDNA-WS_PP) and a plasmid derived from pCDNA3.1 containing the codon-optimized and mutated SARS-COV-2 full-length WS gene (pCTV-WS_PP). Spike (~141 KDa). Β-actin (~48 KDa).

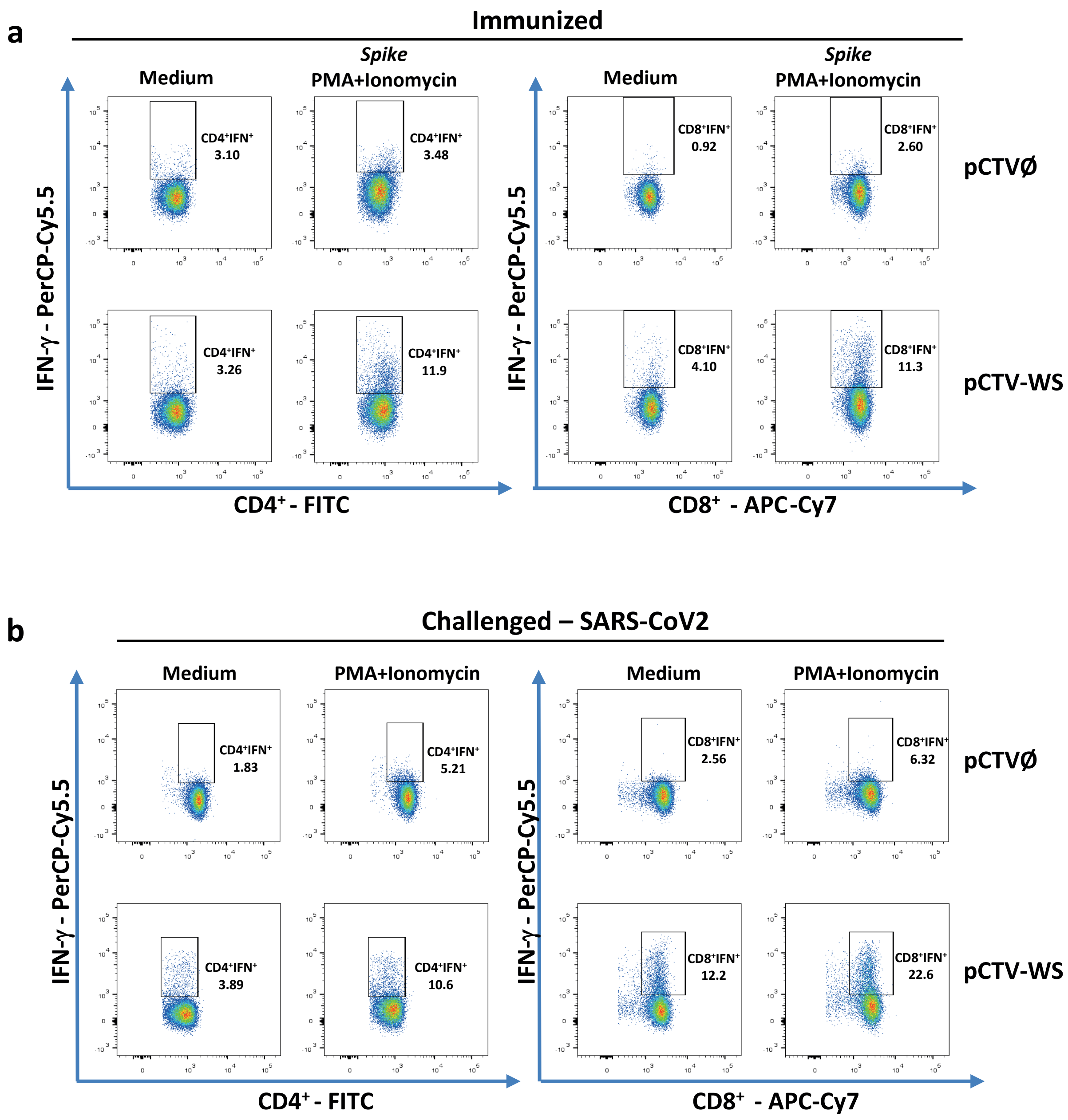

Supplementary Figure 5: Density plots representative of IFN-γ production by CD4$^+$ and CD8$^+$ T cells in spleen samples. (a) Representative density plots showing CD4$^+$ and CD8$^+$ T cells-producing IFN-γ from pCTVØ or pCTV-WS mice restimulated with WS protein and PMA/Ionomycin. **(b)** Representative density plots showing CD4$^+$ and CD8$^+$ T cells-producing IFN-γ from pCTVØ or pCTV-WS mice challenged with Wuhan strain at 5 DPI, stimulated with PMA/Ionomycin [n = 4 mice/group].

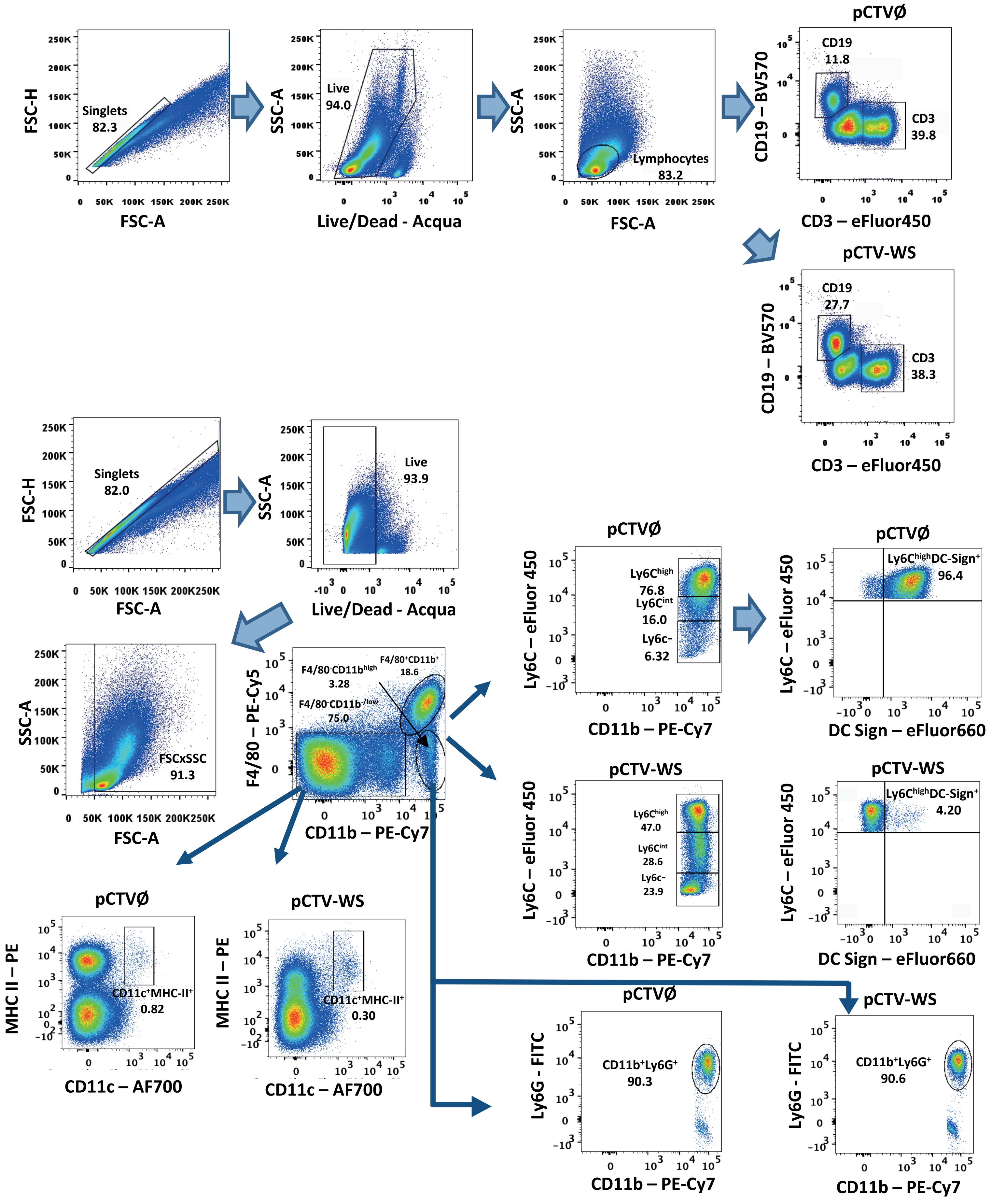

Supplementary Figure 6: Density plots representative of immune cells frequency in lung samples of K18-hACE2 mice at 5DPI. Representative density plots showing gate strategy used for detection of B cells ($CD19^{+}$), T cells ($CD3^{+}$), inflammatory monocytes ($F4/80^{+}CD11b^{+}Ly6C^{high}$), intermediate monocytes ($F4/80^{+}CD11b^{+}Ly6C^{int}$), monocytes ($F4/80^{+}CD11b^{+}Ly6C^{-}$), monocyte-derived dendritic cells ($F4/80^{+}CD11b^{+}Ly6C^{high}DC\text{-}Sign^{+}$), neutrophils ($F4/80^{-}CD11b^{high}Ly6G^{+}$) and classical dendritic cells ($CD11b^{-/low}CD11c^{+}MHC\text{-}II^{+}$) at 5 DPI in lung samples of pCTVØ and pCTV-WS immunized K18-hACE2 mice [n = 4 mice/group].

Supplementary Table 1: qPCR primer sequences

Genes	Primer Sequences	
**β-actin**	F 5’ CGATGCCCTGAGGCTCTTT 3’	R 5’TGGATGCCACAGGATTCCAT 3’
**CCL2**	F 5’ TGGCTCAGCCAGATGCAGT 3’	R 5’TTGGGATCATCTTGCTGGTG 3’
**CCL5**	F 5’ CAAGTGCTCCAATCTTGCAGTC 3’	R 5’TTCTCTGGGTTGGCACACAC 3’
**CXCL9**	F 5’ AATGCACGATGCTCCTGCA 3’	R 5’AGGTCTTTGAGGGATTTGTAGTGG 3’
**CXCL10**	F 5’ GCCGTCATTTTCTGCCTCA 3’	R 5’CGTCCTTGCGAGAGGGATC 3’
**GAPDH**	F 5’ GGCAAATTCAACGGCACAGT 3’	R 5’ AGATGGTGATGGGCTTCCC 3’
**HPRT**	F 5’ GGCAAATTCAACGGCACAGT 3’	R 5’ AGATGGTGATGGGCTTCCC 3’
**IFN-β**	F 5’ CAGCTCCAAGAAAGGACGAAC 3’	R 5’ GGCAGTGTAACTCTTCTGCAT 3’
**IFN-γ**	F 5’ AACGCTACACACTGCATCTTGG 3’	R 5’ GCCGTGGCAGTAACAGCC 3’
**IL-1β**	F 5’ ACCTGTCCTGTGTAATGAAAGACG 3’	R 5’ TGGGTATTGCTTGGGATCCA 3’
**IL-5**	F 5’ AAAGAGAAGTGTGGCGAGGAGA 3’	R 5’ CACCAAGGAACTCTTGCAGGTAA 3’
**IL-6**	F 5’ TGTTCTCTGGGAAATCGTGGA 3’	R 5’ AAGTGCATCATCGTTGTTCATACA 3’
**IL-12 p40**	F 5’ TGGTTTGCCATCGTTTTGCTG 3’	R 5’ ACAGGTGAGGTTCACTGTTTCT 3’
**TNF-α**	F 5’ CCCTCACACTCAGATCATCTTCT 3’	R 5’ GCTACGACGTGGGCTACAG 3’
**E gene**	F 5’ ACAGGTACGTTAATAGTTAATAGCGT3’	R 5’ ATATTGCAGCAGTACGCACACA 3’
	Probe FAM-ACACTAGCCATCCTTACTGCGCTTCG-BBQ	

Supplementary Table 2: Antibodies

Marker	Fluorochrome	Clone	Manufacturer
CD3	eFluor 450	17A2	eBioscience
CD3	FITC	145-2C11	BD Bioscience
CD4	FITC	GK1.5	BD Bioscience
CD4	APC	RM4-5	Biolegend
CD8a	APC-Cy7	53-6.7	Biolegend
CD11b	PE-Cy7	M170	eBioscience
CD11c	Alexa Fluor 700	N418	eBioscience
CD19	BV 570	6D5	Biolegend
DC. SIGN	eFluor 660	MMD3	eBioscience
F4/80	PE-Cy5	BM8	eBioscience
Ly6C	eFluor 450	HK1.4	eBioscience
IFN-γ	PerCP-Cy5.5	XMG1.2	eBioscience
Ly6G	FITC	1A8	eBioscience
MHC II	PE	AF6-120.1	eBioscience
