# Supplementary material for: Differential requirement of neutralizing antibodies and T cells on protective immunity to SARS-CoV-2 variants of concern
Source: NPJ Vaccines. 2023 Feb 13;8:15. doi: 10.1038/s41541-023-00616-y (PMC9923671; doi:10.1038/s41541-023-00616-y)
Supplement: Supplementary file 2 — REPORTING SUMMARY [file 41541_2023_616_MOESM2_ESM.pdf]

nature portfolio

Corresponding author(s): DBPR NPJVACCINES-02460R

Last updated by author(s): Jan 3, 2023

Reporting Summary

Nature Portfolio wishes to improve the reproducibility of the work that we publish. This form provides structure for consistency and transparency in reporting. For further information on Nature Portfolio policies, see our Editorial Policies and the Editorial Policy Checklist.

Statistics

For all statistical analyses, confirm that the following items are present in the figure legend, table legend, main text, or Methods section.

n/a	Confirmed	
☐	☒	The exact sample size (*n*) for each experimental group/condition, given as a discrete number and unit of measurement
☐	☒	A statement on whether measurements were taken from distinct samples or whether the same sample was measured repeatedly
☐	☒	The statistical test(s) used AND whether they are one- or two-sided<br>*Only common tests should be described solely by name; describe more complex techniques in the Methods section.*
☐	☒	A description of all covariates tested
☐	☒	A description of any assumptions or corrections, such as tests of normality and adjustment for multiple comparisons
☐	☒	A full description of the statistical parameters including central tendency (e.g. means) or other basic estimates (e.g. regression coefficient) AND variation (e.g. standard deviation) or associated estimates of uncertainty (e.g. confidence intervals)
☐	☒	For null hypothesis testing, the test statistic (e.g. *F*, *t*, *r*) with confidence intervals, effect sizes, degrees of freedom and *P* value noted<br>*Give P values as exact values whenever suitable.*
☒	☐	For Bayesian analysis, information on the choice of priors and Markov chain Monte Carlo settings
☒	☐	For hierarchical and complex designs, identification of the appropriate level for tests and full reporting of outcomes
☒	☐	Estimates of effect sizes (e.g. Cohen's *d*, Pearson's *r*), indicating how they were calculated

Our web collection on statistics for biologists contains articles on many of the points above.

Software and code

Policy information about availability of computer code

Data collection	Western Blot Images: AI600<br>Flow Cytometry: BD FACSDIVA V8.0.1<br>ELISA:SOFTmaxPRO V4.3.1 LS<br>IFA: Zen
Data analysis	Western Blot Images: ImageJ<br>Flow Cytometry: FlowJo V10.5.3<br>Statistics: GraphPad Prism V7.0<br>IFA: NIS-Elements Viewer

For manuscripts utilizing custom algorithms or software that are central to the research but not yet described in published literature, software must be made available to editors and reviewers. We strongly encourage code deposition in a community repository (e.g. GitHub). See the Nature Portfolio guidelines for submitting code & software for further information.

Data

Policy information about availability of data

All manuscripts must include a data availability statement. This statement should provide the following information, where applicable:

- Accession codes, unique identifiers, or web links for publicly available datasets
- A description of any restrictions on data availability
- For clinical datasets or third party data, please ensure that the statement adheres to our policy

All data generated or analysed during this study are included in this published article and its supplementary information files.

Human research participants

Policy information about studies involving human research participants and Sex and Gender in Research.

Reporting on sex and gender	Not applicable
Population characteristics	Not applicable
Recruitment	Not applicable
Ethics oversight	Not applicable

Note that full information on the approval of the study protocol must also be provided in the manuscript.

Field-specific reporting

Please select the one below that is the best fit for your research. If you are not sure, read the appropriate sections before making your selection.

☒ Life sciences ☐ Behavioural & social sciences ☐ Ecological, evolutionary & environmental sciences

For a reference copy of the document with all sections, see nature.com/documents/nr-reporting-summary-flat.pdf

Life sciences study design

All studies must disclose on these points even when the disclosure is negative.

Sample size	The group sizes for experiments with mice, hamsters and human samples were determined by power calculations statistical analysis.
Data exclusions	No data were excluded from the analysis.
Replication	The experiments were repeated at least twice, if the statistical significance was P<0.05. All attempts at replication were successful.
Randomization	In mice and hamsters experiments we used age and sex-matched individual in the control groups.
Blinding	Except for the histopathology and immunohistochemical description that was performed by two independent observers, we didn't find the need for blinding analysis in the other experiments, because data was objective and phenotypes were very distinct.

Reporting for specific materials, systems and methods

We require information from authors about some types of materials, experimental systems and methods used in many studies. Here, indicate whether each material, system or method listed is relevant to your study. If you are not sure if a list item applies to your research, read the appropriate section before selecting a response.

Materials & experimental systems

n/a	Involved in the study	
☐	☒	Antibodies
☐	☒	Eukaryotic cell lines
☒	☐	Palaeontology and archaeology
☐	☒	Animals and other organisms
☒	☐	Clinical data
☒	☐	Dual use research of concern

Methods

n/a	Involved in the study	
☒	☐	ChIP-seq
☐	☒	Flow cytometry
☒	☐	MRI-based neuroimaging

Antibodies

Antibodies used

- Rat anti-mouse eFluor450 CD3 eBioscience Clone: 17A2; Cat. # 48-0032-82; Lot: E08480-1631.
- Hamster Anti-Mouse FITC CD3e BD Bioscience Clone: 145-2C11; Cat. # 553062; Lot: 24261.
- Rat anti-mouse CD4a BioXCell Clone: GK1.5; Cat. # BE0003-1; Lot: 607516J3.
- Rat anti-mouse FITC CD4 BD Bioscience Clone: GK1.5; Cat. # 553729; Lot: 11783.
- Rat anti-mouse APC CD4 Biolegend Clone: RM4-5; Cat. # 100516; Lot: B170521.
- Rabbit anti-mouse CD4 Abcam Clone: EPR19514; Cat. # ab183685; Lot: GR3375645-15.
- Rat anti-mouse CD8a BioXCell Clone: 2.43; Cat. # BE0061; Lot: 62461601.
- Rat anti-mouse APC-Cy7 CD8a Biolegend Clone: 53-6.7; Cat. # 100714; Lot: B217172.
- Rabbit anti-mouse CD3 Abcam Clone: EPR21769; Cat. # ab217344; Lot: GR3366917-15.
- Rat anti-mouse PE-Cy7 CD11b eBioscience Clone: M170; Cat. # 25-0112-82; Lot: 1956821.
- Hamster Anti-Mouse Alexa Fluor 700 CD11c eBioscience Clone: N418; Cat. # 56-0114-82; Lot: 4300335.
- Rat anti-mouse BV 570 CD19 Biolegend Clone: 6D5; Cat. # 115535; Lot: B158670.
- Rabbit anti-mouse CD19 Abcam Clone: EPR23174-145; Cat. # ab245235; Lot: GR3357580-6.
- Mouse anti-mouse eFluor 660 CD209a eBioscience Clone: MMD3; Cat. # 50-2094-82; Lot: E16337-103.
- Rat anti-mouse PE-Cy5 F4/80 eBioscience Clone: BM8; Cat. # 15-4801-82; Lot: 1953010.
- Rat anti-keyhole (KLH) IgG2b BioXCell Clone: LTF-2; Cat. # BP0090; Lot: 629816D1.
- Rat anti-mouse eFluor450 LY6C eBioscience Clone: HK1.4; Cat. # 48-5932-82; Lot: E14323-103.
- Rat anti-mouse PerCP-Cy5.5 IFN- eBioscience Clone: XMG1.2; Cat. # 45-7311-82; Lot: E08426-1319.
- Rat anti-mouse FITC LY6G eBioscience Clone: 1A8; Cat. # 11-9668-80; Lot: 2012522.
- Mouse anti-mouse PE MHC II eBioscience Clone: AF6-120.1; Cat. # 12-5320-82; Lot: E19826-102.
- Goat anti-mouse IgG-HRP SoutherBiotech Cat. # 1030-05; Lot: B1411-PD01B
- Goat anti-mouse IgG1-HRP SoutherBiotech Cat. # 1070-05; Lot: J6908-T229B
- Goat anti-mouse IgG2c-HRP SoutherBiotech Cat. # 1079-05; Lot: K5210-P951O
- Goat anti-hamster IgG(H+L)-HRP SoutherBiotech Cat. # 6060-05; Lot: I4711-Z990B
- Goat anti-hamster IgG1-HRP SoutherBiotech Cat. # 1940-05; Lot: H2218-ZD60B
- Goat anti-hamster IgG2/IgG3-HRP SoutherBiotech Cat. # 1935-05; Lot: C8110-VP60C
- SARS-CoV-2 Spike RBD Sino Biologicals Cat. # 40592-T62; Lot: HD14AU0608

Validation

Each antibody was validated following manufacturer's instructions or based on previously published methods. The antibodies were titrated to obtain the optimal concentration for use in our panels. The antibody specificity was compared to isotype control when applicable.

Eukaryotic cell lines

Policy information about cell lines and Sex and Gender in Research

Cell line source(s)

HEK293 (ATCC CRL-1573)
Vero E6 (ATCC CRL-1586)

Authentication

Cells were low passage cells from ATCC, which authenticates them. Cell morphology and growth was consistent with HEK293 and Vero-E6.

Mycoplasma contamination

All cell lines tested negative for mycoplasma contamination

Commonly misidentified lines
(See ICLAC register)

Not applicable

Animals and other research organisms

Policy information about studies involving animals; ARRIVE guidelines recommended for reporting animal research, and Sex and Gender in Research

Laboratory animals

C57BL/6: 6-10 weeks old
K18-hACE2: 6-10 weeks old
K18-hACE2/B-KO: 6-10 weeks old
Golden Syrian hamsters: 6-10 weeks old

Wild animals | Not applicable

Reporting on sex | Not applicable

Field-collected samples | The study did not involve samples collected from the field

Ethics oversight | Protocols were approved by the Committee on Ethics in the Use of Animals (CEUA) of Fundação Oswaldo Cruz (CEUA protocol LW25/20) and the Universidade de São Paulo (CEUA protocol 105/2020)

Note that full information on the approval of the study protocol must also be provided in the manuscript.

Flow Cytometry

Plots

Confirm that:

☒ The axis labels state the marker and fluorochrome used (e.g. CD4-FITC).

☒ The axis scales are clearly visible. Include numbers along axes only for bottom left plot of group (a 'group' is an analysis of identical markers).

☒ All plots are contour plots with outliers or pseudocolor plots.

☒ A numerical value for number of cells or percentage (with statistics) is provided.

Methodology

Sample preparation

Splenocytes were isolated by spleen maceration using a 100 µm pore cell strainer (Cell Strainer, BD Falcon). Then, erythrocytes were lysed with ammonium-chloride-potassium (ACK) buffer and the cell number was adjusted to 2x10^6 cells/well. for intracellular staining of IFN-g. Splenocytes from immunized mice were stimulated with 10 µg/mL of WS protein for 24h and in the last 4h it was added PMA (50ng/mL), Ionomycin (500ng/uL), Golgi Plug (BD Bioscience) and Stop Golgi (BD Bioscience). Splenocytes from challenged mice were stimulated only with PMA and Ionomycin for 4h in the presence of Golgi Plug and Stop Golgi. Cells were stained with Live/Dead stain (Acqua, Invitrogen) for 20 min at 4ºC in the dark, incubated with FcBlock (BD Bioscience) for 20 min at 4ºC and then stained with anti-CD3 (eFLuor405, eBioscience), anti-CD4 (Fitc, BD Bioscience), anti-CD8 (APC-Cy7, Biolegend). Cells were permeabilized with Cytofix/Cytoperm (BD Bioscience) for 20 min at 4ºC in the dark and incubated with anti-IFN-g (PerCp-Cy5.5, eBioscience) for 30 min at 4ºC.

Mice lungs were perfused with cold PBS, collected, digested with 100µg/mL liberase (Roche) and incubated at 37ºC for 30 minutes for cell dissociation. Cells were filtered using a 100 µm pore cell strainer (Cell Strainer, BD Falcon) and purified using a 30% Percoll gradient. Erythrocytes were lysed with ammonium-chloride-potassium (ACK) buffer and the cell number was adjusted to 2x10^6 cells/well. Cells were stained with Live/Dead stain (Acqua, Invitrogen) for 20 min at 4ºC in the dark, incubated with FcBlock (BD Bioscience) for 20 min at 4ºC and then stained with anti-CD3 (eFLuor405, eBioscience), anti-CD4 (APC, Biolegend), anti-CD8 (APC-Cy7, Biolegend), anti-CD19 (BV570, Biolegend), anti-CD11b (PE-Cy7, eBioscience), CD11c (AF700, eBioscience), DC-Sign (eFluor660, eBioscience), F4/80 (PE-Cy5, eBioscience), Ly6C (eFluor405, eBiscience), Ly6G (FITC, eBioscience) and MHC II (PE, CD Bioscience).

Instrument | BD LSRFortessa

Software | Acquisition: BD FACSDIVA V8.0.1
Data analysis: FlowJo v10.5.3

Cell population abundance | All CD3, CD4, CD8 and CD19. All monocytes and neutrophils.

Gating strategy

For B and T cells, we started gate strategy by single cells (FSC-A x FSC-H) with a gate in the diagonal, followed by live cells (Live/Dead x SSC-A) with a gate in the negative population for Live/Dead stain. After that we made a gate in lymphocyte region (FSC-A x SSC-A). Next, we used the dot plot CD3 x CD19 to separated T (positive only for CD3) and B cells (positive only for CD19).
For myeloid cells, we started gate strategy by single cells (FSC-A x FSC-H) with a gate in the diagonal, followed by live cells (Live/Dead x SSC-A) with a gate in the negative population for Live/Dead stain. After that we made a gate excluding debris (FSC-A x SSC-A). Next, we used the dot plot CD11b x F4/80 to separated 3 different populations: F4/80+CD11b+ (double positive), F4/80-CD11b High (positive only for CD11b) and F4/80-CD11b-/Low (double negative and with low expression of CD11b).
Inside the gate for F4/80+CD11b+ double positive cells, we performed new gates, separating 3 subpopulations: Inflammatory monocytes (F4/80+CD11b+Ly6C+), intermediate monocytes (F4/80+CD11b+Ly6C Int) and monocytes (F4/80+CD11b+Ly6C-).
For MO-DC cells, a new gate was made inside inflammatory population, being positive for DC-Sign (F4/80+CD11b+Ly6C High DC-Sign+).
Neutrophils were gated inside the population positive only for CD11b (F4/80-CD11b High), being positive for Ly6G (F4/80-CD11b High Ly6G+).
Classical dendritic cells were gated inside F4/80-CD11b-/Low, being double positive for CD11c and MHC-II (F4/80-CD11b-/Low CD11c+MHC-II+).

Gate strategy are presented in Fig. S5.

☒ Tick this box to confirm that a figure exemplifying the gating strategy is provided in the Supplementary Information.
